# A group B streptococcal type VII-secreted LXG toxin mediates interbacterial competition and colonization of the murine female genital tract

**DOI:** 10.1128/mbio.02088-24

**Published:** 2024-08-27

**Authors:** Alyx M. Job, Kelly S. Doran, Brady L. Spencer

**Affiliations:** 1 Department of Immunology and Microbiology, University of Colorado Anschutz, Aurora, Colorado, USA; Universite de Geneve, Geneva, Switzerland

**Keywords:** *Streptococcus agalactiae*, group B *Streptococcus*, GBS, type VII secretion system, LXG toxins, interbacterial competition, vaginal colonization

## Abstract

Group B *Streptococcus* (GBS) asymptomatically colonizes the vagina but can opportunistically ascend to the uterus and be transmitted vertically during pregnancy, resulting in neonatal pneumonia, bacteremia, and meningitis. GBS is a leading etiologic agent of neonatal infection and understanding the mechanisms by which GBS persists within the polymicrobial female genital mucosa has the potential to mitigate subsequent transmission and disease. Type VIIb secretion systems (T7SSb) are encoded by Bacillota and often mediate interbacterial competition using LXG toxins that contain conserved N-termini important for secretion and variable C-terminal toxin domains that confer diverse biochemical activities. Our recent work characterized a role for the GBS T7SSb in vaginal colonization and ascending infection but the mechanisms by which the T7SSb promotes GBS persistence in this polymicrobial niche remain unknown. Herein, we investigate the GBS T7SS in interbacterial competition and GBS niche establishment in the female genital tract. We demonstrate GBS T7SS-dependent inhibition of mucosal pathobiont *Enterococcus faecalis* both *in vitro* using predator-prey assays and *in vivo* in the murine genital tract and found that a GBS LXG protein encoded within the T7SS locus (herein named group B streptococcal LXG Toxin A) contributes to these phenotypes. We identify BltA as a T7SS substrate that is toxic to *E. coli* and *S. aureus* upon induction of intracellular expression along with associated chaperones. Finally, we show that BltA and its chaperones contribute to GBS vaginal colonization. Altogether, these data reveal a role for a novel T7b-secreted toxin in GBS mucosal persistence and competition.

**IMPORTANCE:**

Competition between neighboring, non-kin bacteria is essential for microbial niche establishment in mucosal environments. Gram-positive bacteria encoding T7SSb have been shown to engage in competition through the export of LXG-motif-containing toxins, but these have not been characterized in group B *Streptococcus* (GBS), an opportunistic colonizer of the polymicrobial female genital tract. Here, we show a role for GBS T7SS in competition with mucosal pathobiont *Enterococcus faecalis*, both *in vitro* and *in vivo*. We further find that a GBS LXG protein contributing to this antagonism is exported by the T7SS and is intracellularly toxic to other bacteria; therefore, we have named this protein group B streptococcal LXG Toxin A (BltA). Finally, we show that BltA and its associated chaperones promote persistence within female genital tract tissues, *in vivo*. These data reveal previously unrecognized mechanisms by which GBS may compete with other mucosal opportunistic pathogens to persist within the female genital tract.

## INTRODUCTION

Interbacterial competition is necessary for bacterial niche establishment, particularly in polymicrobial mucosal environments within the host (1). Bacteria utilize numerous systems to compete with their non-kin neighbors, including nutrient restriction, production of bacteriocins or antibiotics, as well as secretion systems that facilitate the export of toxic effectors (2). The Type I, Type IV, and Type VI Secretion systems in Gram-negative bacteria have been particularly well studied for their roles in interbacterial competition by secreted effectors (3). However, mechanisms of interbacterial antagonism by specialized secretion systems in Gram-positive bacteria have been less characterized.

Type VIIb secretion systems are encoded by Bacillota and are comprised of core machinery proteins (membrane-bound EsaA, EssA, EssB, and cytoplasmic EsaB) as well as a membrane-associated FtsK/SpoIII ATPase, EssC, which drives the export of small ɑ-helical proteins and effectors (4). WXG100 proteins (named for their central Tryptophan-X-Glycine motif and 100 amino acid structure) are canonical substrates of the T7SS and have been implicated in virulence in numerous Gram-positive species (5–7). LXG toxins are also common substrates of the T7SSb and are characterized by ɑ-helical N-terminal structures containing a Leu-X-Gly motif. These N-terminal domains have been shown to interact with adjacently encoded ɑ-helical chaperones or LXG-associated ɑ-helical proteins (Laps), and this complex is hypothesized to be important for recognition and trafficking to the secretion machinery (4, 8). The C-terminal domain of these proteins often confers toxic activity and its structure is variable between LXG proteins depending on their biochemical function (4). Indeed, LXG toxins across and within genera and species are often biochemically diverse and the while functions of many remain unknown, the few that have been studied may act as nucleases, NADases, or phospholipases (4, 9, 10). In several Gram-positive bacteria, T7SSb-secreted LXG toxins have been shown to exhibit toxicity and/or contribute to interbacterial competition (8, 9, 11–15), and T7SSb has been hypothesized to have evolved for interbacterial antagonism, promoting niche establishment by both commensals and pathogens (16). Therefore, the study of these toxins across additional T7SSb-encoding opportunistic pathogens is needed to better understand the mechanisms of competition in the mucosa.

*Streptococcus agalactiae* or group B *Streptococcus* (GBS) is a Gram-positive pathogen that asymptomatically colonizes the vaginal tract but causes infection upon ascension to higher genital tract tissues, such as the uterus (17). During pregnancy, GBS vaginal colonization and ascending infection can result in adverse pregnancy outcomes (including stillbirth, pre-term birth, and chorioamnionitis) as well as neonatal pneumonia, bacteremia, and meningitis upon vertical transmission (18). As vaginal colonization is a prerequisite for the development of early-onset GBS disease in infants (19), a better understanding of the bacterial factors that promote the persistence of GBS within the vaginal mucosa is needed. While the microbiota often confers colonization resistance in mucosal niches (20, 21), GBS is not only capable of establishing persistent colonization but is also known to perturb the vaginal microbiota during colonization (21–23). Thus, the specific mechanisms by which GBS competes with the microbiota and/or mucosal pathobionts to persist within this polymicrobial niche warrant further study.

We previously characterized the GBS T7SSb and identified four T7SS subtypes across GBS clinical isolates based on variation within the C-terminus of the EssC ATPase and unique downstream repertoires of putative effectors (24). Despite this effector diversity across subtypes, three of the four GBS T7SSb subtypes’ loci display a conserved synteny downstream of the T7SS machinery genes, encoding for two putative chaperones and a putative LXG toxin, similar to those described in other Gram-positive species (8, 25). We demonstrated that the highly prevalent subtype I GBS T7SS contributes to vaginal colonization and ascending infection, *in vivo* (7, 24). However, the mechanism by which the subtype I T7SS establishes this niche has not been determined. We hypothesize that the GBS T7SSb and, specifically, secreted LXG toxins may promote interbacterial antagonism, facilitating persistent GBS vaginal colonization and infection. Herein, we identify a role for the GBS T7SS in interbacterial competition with *Enterococcus faecalis* both *in vitro* and *in vivo* in the female genital tract. We further identify a GBS LXG protein encoded within the T7SS subtype I locus that contributes to these phenotypes, which we have named the group B streptococcal LXG Toxin A (BltA). We demonstrate that BltA is secreted by the T7SS and is toxic to other bacteria when intracellularly co-expressed with associated protein chaperones. Finally, we show that BltA and its associated chaperones contribute to GBS vaginal colonization and ascending infection. Altogether, our findings indicate a role for T7SS toxin-mediated interbacterial competition in GBS mucosal persistence.

## RESULTS

### GBS subtype I T7SSb contributes to interbacterial competition

**Fig 1:**
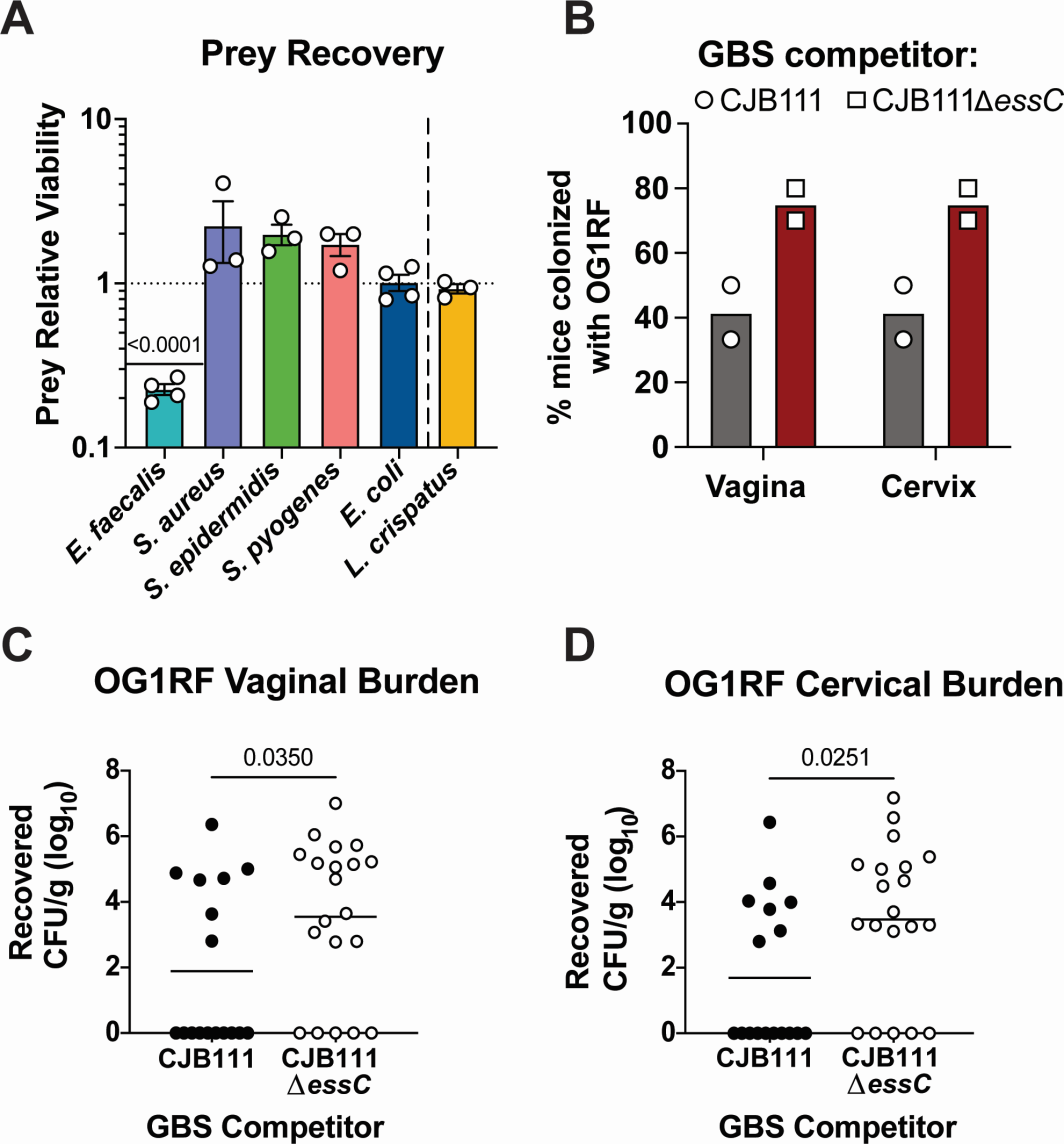
The GBS T7SS reduces recovery of *E. faecalis*, *in vitro* and *in vivo*. (A) Predator-prey inter-species competition experiments were performed in a liquid medium between parental CJB111 GBS or CJB111∆*essC* mutant predator strains and a panel of representative “prey” species. Relative abundance of a given prey strain was calculated as a ratio of prey CFU recovered following 24-hour co-culture with the parental CJB111 predator strain compared to the ∆*essC* predator strain. Statistics reflect one-sample *t*-tests against a hypothetical value of 1. Data represent the mean of three or four independent experiments and error bars represent the standard error of the mean. (B–D) GBS T7SS-mediated inhibition of *E. faecalis* OG1RF during murine vaginal colonization. Either parental CJB111 GBS or the CJB111∆*essC* mutant was co-inoculated with prey *E. faecalis* OG1RF directly into the vaginal lumen (5 × 10^6^ CFU each strain). (B) Percent OG1RF colonization of vaginal or cervical tissue plotted for each of two independent experiments (Bars represent the median; Fisher’s exact test, *P* = 0.0498 in both vaginal and cervical tissue). Recovered *E. faecalis* CFU counts from the (**C**) vaginal and (**D**) cervical tissues of co-colonized mice. Each dot represents one mouse and data from two independent experiments are combined in these figures (*n* = 17, 20 total in parental CJB111 and CJB111∆*essC* mutant co-colonization groups, respectively). The bars in these plots show the median and statistics represent the Mann-Whitney U test.

GBS T7SS promotes persistence within the female genital tract tissues, but it remained unclear whether the GBS T7SS may target other bacteria during niche establishment. The contribution of the T7SSb to interbacterial interactions has been demonstrated in a few Gram-positive organisms, with the breadth and specificity of antagonism differing across genera and species. For example, the *S. intermedius* T7SS has been shown to inhibit a broad range of Gram-positive bacteria (13), while the *E. faecalis* T7SS inhibited only a subset of Gram-positive strains tested (26) and *S. aureus* T7SS inhibition was not observed against *S. epidermidis* (27). To evaluate the ability of GBS to compete with other bacterial species, we grew pure cultures of parental or ∆*essC* mutant CJB111 (predators) and a panel of non-kin bacteria (prey) commonly co-isolated with GBS from human polymicrobial environments, such as the diabetic wound and the vaginal tract. Specified predator and prey bacteria were mixed in a defined ratio and co-cultured statically in liquid media for 24 hours before assessing relative prey viability (13, 26, 28). Similar to previous T7SSb studies performed in other Gram-positive species, we did not observe T7SSb-mediated antagonism against Gram-negative organism, *Escherichia coli* (Fig. 1A). The CJB111 T7SS also did not inhibit the growth of Gram-positive species *Staphylococcus aureus*, *Staphylococcus epidermidis*, *Streptococcus pyogenes*, or *Lactobacillus crispatus*. We did, however, observe a significant decrease in *Enterococcus faecalis* (representative strain OG1RF) CFU recovered in the presence of the parental CJB111 compared to the CJB111∆*essC* mutant. This GBS competition with *E. faecalis*, but not with other Gram-positive species such as *S. aureus*, was also demonstrated in the CNCTC 10/84 clinical isolate background, which expresses a subtype III T7SS locus (Fig. S1A). These competition phenotypes were not due to differences in GBS burdens, as the recovered parental GBS and ∆*essC* mutant CFU were equivalent in any given predator-prey competition pair (Fig. 1SB, C, and S2A). Finally, to determine the breadth of the GBS T7SS target range, we performed competition with CJB111 and CJB111∆*essC* GBS against a panel of 15 additional *E. faecalis* isolates (see Table S1) (29, 30). Of the *E. faecalis* isolates investigated, all but one were significantly inhibited by GBS in a T7SS-specific manner (Fig. S1D). Interestingly, an isolate of a closely related species, *Enterococcus faecium*, was not inhibited by GBS T7SS. These data suggest that the GBS T7SS may broadly inhibit *E. faecalis* but not all *Enterococcus* species.

*E. faecalis* is also a vaginal opportunistic pathogen, and our laboratory has previously shown that *E. faecalis* is capable of persisting in murine models of vaginal colonization and ascending infection (31). As the vaginal tract is a common niche for both GBS and *E. faecalis*, we hypothesized that this mucosal environment may represent a physiologically relevant site of competition between these opportunistic pathogens. To determine whether GBS can compete with *E. faecalis* OG1RF in this complex environment, we performed *in vivo* co-colonization experiments in which *E. faecalis* was vaginally co-inoculated with either parental CJB111 or the isogenic CJB111∆*essC* mutant in conventional mice. *E. faecalis* OG1RF clears more quickly from the murine vaginal tract compared to GBS CJB111. Therefore, we assessed OG1RF tissue burdens at early colonization time points, at which point parental and ∆*essC* mutant GBS burdens remained equal (Fig. S2B and C) to eliminate differential GBS clearance as a variable in these studies. We found that a higher percentage of mice harbored vaginal and cervical OG1RF when co-colonized with the CJB111∆*essC* mutant compared to the parental CJB111 (Fig. 1B). We further recovered more *E. faecalis* from vaginal and cervical tissue in mice co-colonized with the CJB111∆*essC* mutant compared to mice co-colonized with parental CJB111 (Fig. 1C and D). Collectively, these data suggest a potential role for the GBS T7SS in competition with another opportunistic pathogen within the physiologically relevant niche of the female genital tract.

### LXG toxin BltA contributes to competition with *E. faecalis*

**Fig 2:**
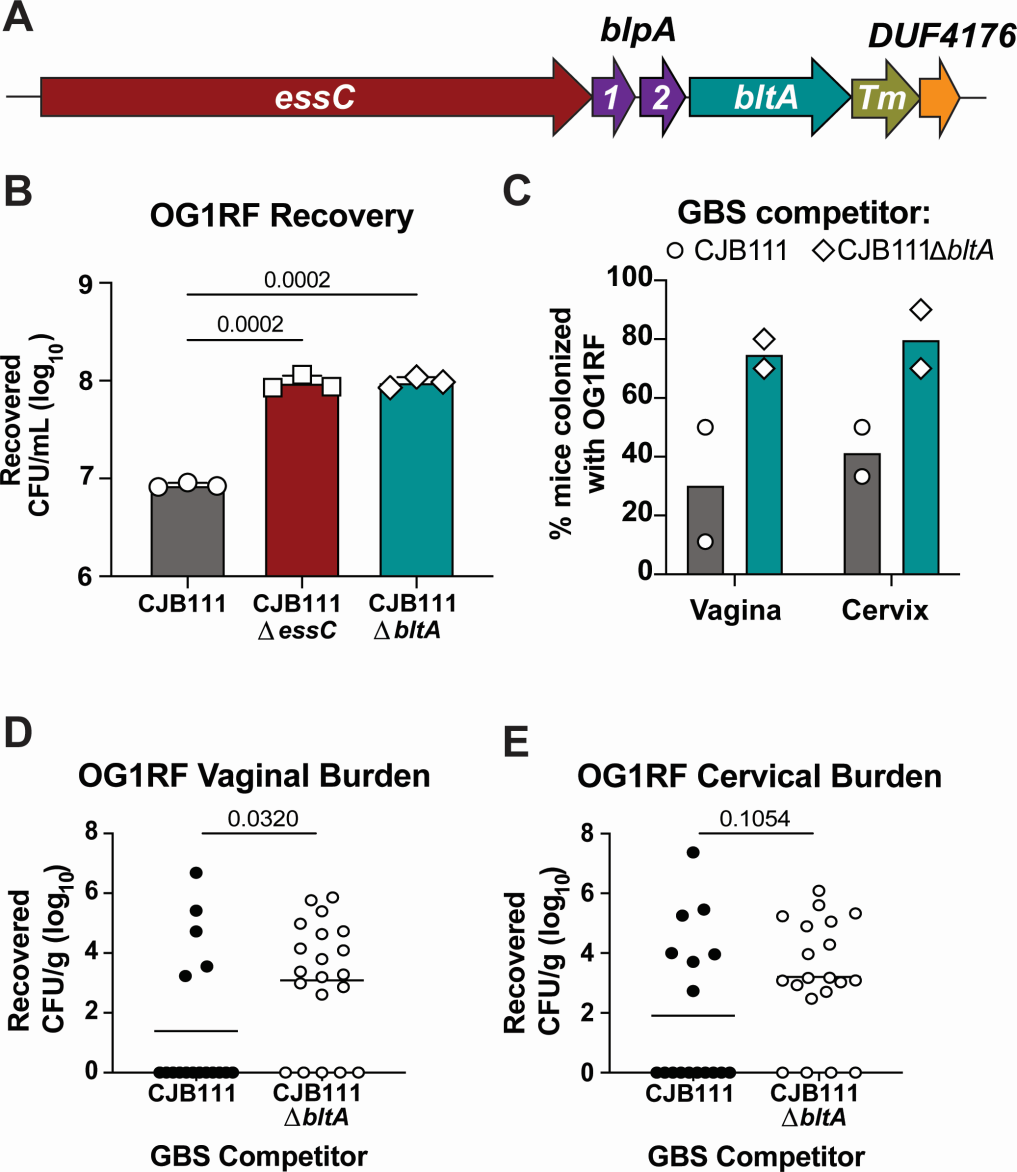
GBS LXG protein BltA contributes to *E. faecalis* antagonism *in vitro* and *in vivo*. (A) Diagram of part of the GBS T7SS subtype I locus. GBS T7SS loci encode an EssC ATPase (*essC*), which drives effector export. The subtype I “LXG module” downstream of *essC* includes genes encoding for putative chaperones (BlpA1-2, purple), an LXG toxin (BltA, teal), a Tm protein (olive green), and a DUF4176 protein (orange). (B) Recovered CFU/mL of *E. faecalis* OG1RF prey following predator-prey inter-species competition experiments performed in liquid medium with parental CJB111 GBS, CJB111∆*essC*, or CJB111∆*bltA* predator strains. Statistics reflect ordinary one-way ANOVA with Tukey’s multiple comparison test. Data represent the mean of three independent experiments and error bars represent the standard deviation. (C–E) BltA-mediated inhibition of *E. faecalis* during murine vaginal colonization. Either parental CJB111 GBS or the CJB111∆*bltA* mutant strain was co-inoculated with prey *E. faecalis* OG1RF directly into the vaginal lumen (5 × 10^6^ CFU each strain). (C) Percent OG1RF colonization of vaginal or cervical tissue plotted for each of two independent experiments (Bars represent the median; Fisher’s exact test, *P* = 0.0086 in vaginal tissue and *P* = 0.0210 in cervical tissue). Recovered *E. faecalis* CFU counts from the (D) vaginal and (E) cervical tissues of co-colonized mice. Each dot represents one mouse and data from two independent experiments are combined in these figures (*n* = 17, 20 total in parental CJB111 and CJB111∆*bltA* mutant co-colonization groups, respectively). The bars in these plots show the median and statistics represent the Mann-Whitney U test.

In other T7SSb^+^ Gram-positive species, LXG toxins have been shown to contribute to the inhibition of other bacteria. In our previous work, we bioinformatically characterized GBS T7SS loci and found that four subtypes exist that encode for unique putative effector repertoires (24). Downstream of the secretion machinery genes, GBS T7SS subtypes I-III loci encode “modules” containing putative *lxg* genes as well as two putative chaperone-encoding genes (Fig. 2A; also known as LXG-associated ɑ-helical proteins; herein called group B streptococcal LXG-associated Proteins, or Blp), which in other species have been shown to facilitate LXG toxin stability and/or secretion (8, 32, 33). Previously, using AlphaFold Multimer modeling, we found that the GBS T7SS subtype I putative chaperones, herein named BlpA1 and BlpA2, are predicted to interact with the putative subtype I LXG toxin, herein named BltA (24). Given the conserved arrangement and function of this module across species, we hypothesized that BltA may promote GBS T7SS interbacterial competition. To assess this, we repeated CJB111 GBS-OG1RF *E. faecalis* predator-prey assays using parental CJB111 and an isogenic CJB111∆*bltA* mutant. Similar to the ∆*essC* mutant, higher levels of *E. faecalis* were recovered in the presence of the ∆*bltA* mutant compared to the presence of parental CJB111 (Fig. 2B), with the GBS CFU recovery remaining equal across these conditions (Fig. S3A). As toxins are commonly encoded adjacent to immunity proteins to prevent self-intoxication, we also performed predator-prey competition assays in which the prey OG1RF expressed the Tm-encoding gene downstream of *bltA* (*ID870_4220*, referred to as 4220) or an empty vector control. Interestingly, we observed GBS T7SS-dependent antagonism of *E. faecalis* regardless of 4220 expression (Fig. S3B). To determine whether BltA may also contribute to the antagonism against *E. faecalis in vivo,* we repeated the vaginal co-colonization experiments described above, this time co-inoculating either parental CJB111 or the isogenic ∆*bltA* mutant with *E. faecalis* into the vaginal tract. Similar to the results with the ∆*essC* mutant above, we observed that more mice co-colonized with the ∆*bltA* mutant harbored OG1RF within the vagina and cervix (Fig. 2C) and at higher tissue burdens (Fig. 2D and E) compared to the group co-colonized with parental CJB111. Reciprocally, GBS tissue burdens at this early colonization timepoint did not significantly differ between the groups (Fig. S3C and D). These data suggest that BltA may contribute to GBS T7SS-mediated interbacterial antagonism against *E. faecalis*.

### BltA is a secreted substrate of the GBS T7SSb subtype I

**Fig 3:**
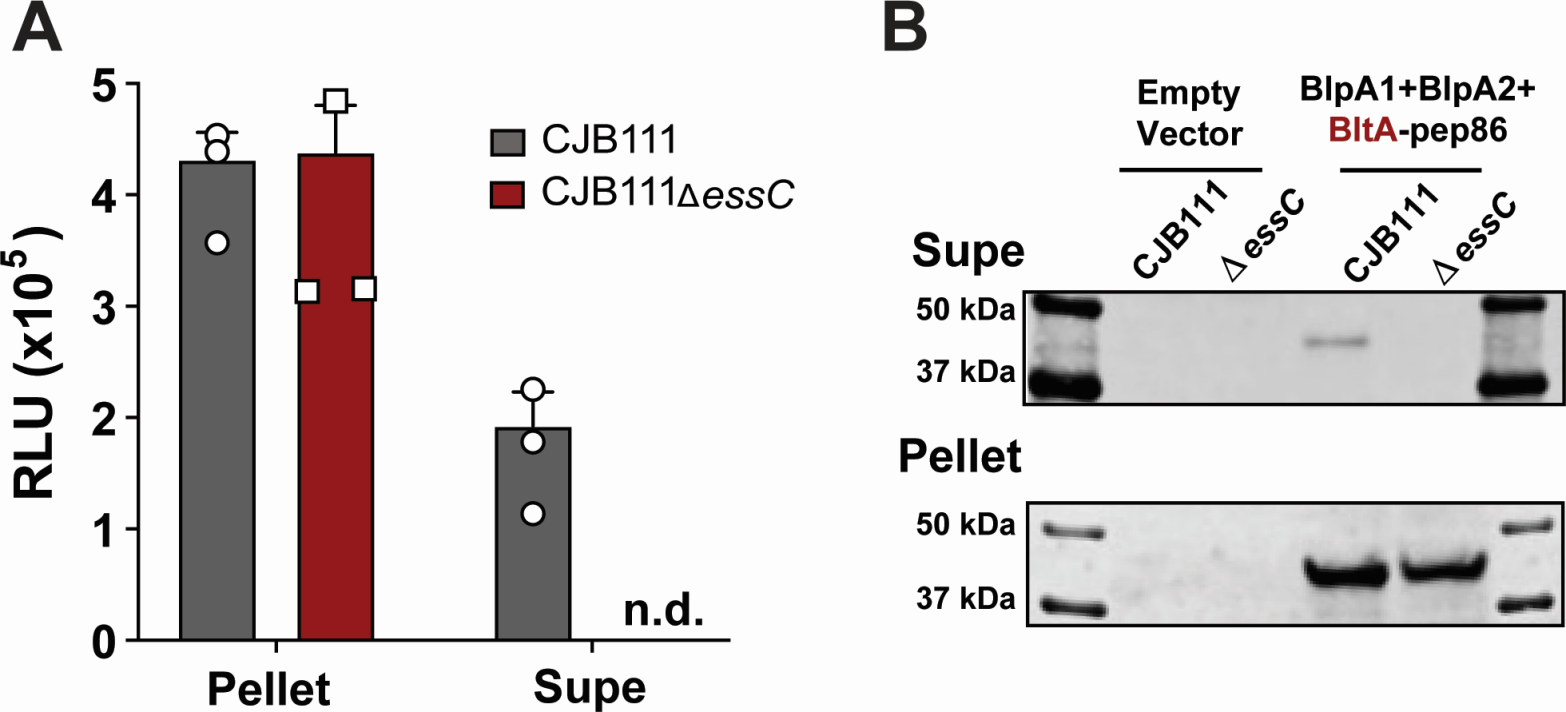
BltA is secreted by the GBS subtype I T7SS in an EssC-dependent manner. (**A**) BltA-pep86 was co-expressed with BlpA1-2 chaperones in *trans* in CJB111 or the CJB111∆*essC* mutant and luminescence (relative light units, RLU) was detected in cell-associated lysates, and cell-free supernatant fractions after the addition of the large nanoluciferase 11S fragment and substrate. Data in panel A represent the mean of three independent experiments and error bars represent the standard deviation. (**B**) Western blot showing EssC-dependent secretion of BltA from subtype I strain CJB111. The blot pictured is representative of three independent experiments. Coomassie-stained gels can be found as loading controls for supernatant and cell-associated pellet fractions in Fig. S4B.

The *bltA* gene is encoded downstream of the T7SSb machinery genes and adjacent to putative chaperone-encoding genes (*blpA1-2*), a region that typically encodes for T7SSb substrates. To evaluate whether BltA is secreted by the GBS T7SSb, we utilized a Nanoluciferase-based secretion assay (Nanobit) optimized to identify T7SS effectors in *S. aureus* (32, 34, 35). Briefly, the BltA C-terminus was tagged with the small Nanoluciferase subunit pep86 and co-expressed with putative chaperones BlpA1-2 in *trans* in parental strain CJB111 or in the CJB111∆*essC* mutant. Secretion of BltA-pep86 was then evaluated by supplementing stationary phase cell lysates or corresponding cell-free supernatants with the large Nanoluciferase 11S subunit (to reconstitute the enzyme) and its substrate and measuring the resulting bioluminescence. Bioluminescent signal exceeding that of the corresponding parental CJB111 or CJB111∆*essC* mutant empty vector controls (lacking pep86) was reported as relative light units (RLU). While we observed similar luminescence signals from both CJB111 and CJB111∆*essC* cell-associated lysate fractions, we only observed luminescence from parental CJB111 supernatant, not from CJB111∆*essC* supernatant (Fig. 3A). To confirm BltA secretion by the GBS T7SS, we probed for BltA within these cell lysates and supernatant using an anti-pep86 antibody (Promega). As a control, we also assessed the secretion of the canonical T7SS substrate EsxA, which we demonstrated previously to be secreted in an EssC-dependent manner in GBS (Fig. S4A) (7). Similar to the Nanobit assay, we observed EssC-dependent BltA secretion by Western blot (Fig. 3B) and did not detect any signal in the cell associated or supernatant of the empty vector control strains (which lack the pep86 tag). Collectively, these data indicate that BltA is indeed a GBS T7SSb subtype I substrate.

Finally, secretion of T7SS substrates has been shown to be interdependent (36) but it is unknown whether export of known GBS T7SS substrate EsxA is impacted by the presence or absence of other T7SS effectors. To determine whether loss of BlpA1-2 or BltA impact EsxA secretion, we assessed extracellular EsxA levels in cell-free supernatant. We detected similar levels of EsxA in the cell-associated pellet and supernatant fractions of ∆*blpA1-2 and* ∆*bltA* mutants compared to that of parental CJB111, indicating that neither GBS BltA nor its associated chaperones BlpA1-2 are necessary for EsxA T7SS export (Fig. S4C).

### Intracellular BltA expression intoxicates bacteria in a chaperone-dependent manner

**Fig 4:**
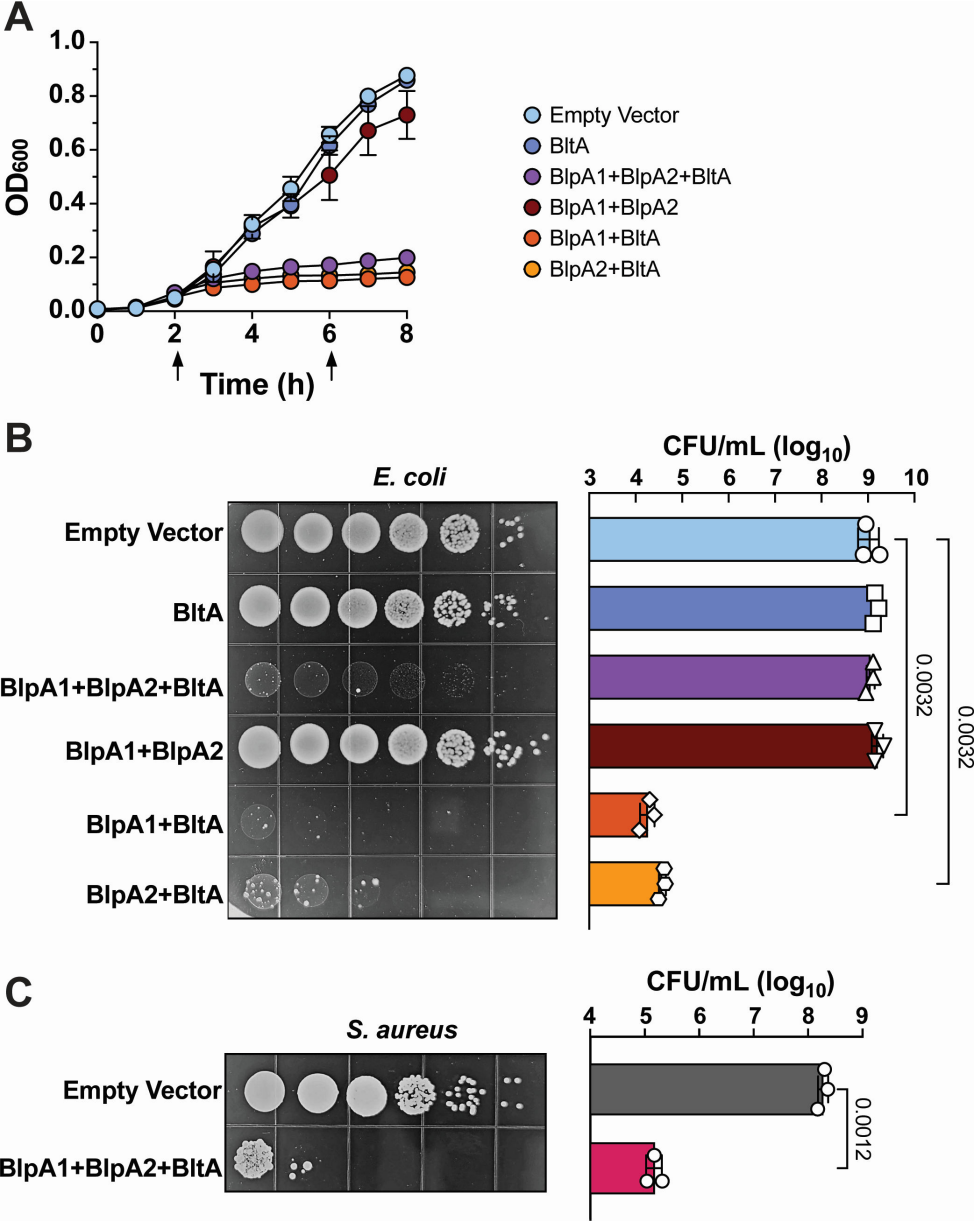
Inducible intracellular expression of BltA with associated chaperones inhibits growth of *E. coli* and *S. aureus*. (**A**) Growth of *E. coli* in liquid medium (as measured by OD_600_) expressing rhamnose-inducible plasmids containing an empty vector, BltA alone, BlpA1 + BlpA2 + BltA, BlpA1 + BlpA2 alone, BlpA1 + BltA, or BlpA2 + BltA. In panel A, arrows indicate the addition of rhamnose to induce protein expression. Data in panel A represent the mean of three independent experiments and error bars represent the standard error of the mean. (**B**) Viability of the above *E. coli* strains serially diluted and plated on rhamnose-containing media. Serial dilutions are shown in the representative image on the left and quantified in the panel on the right. (**C**) Viability of *S. aureus* strains expressing an empty vector or an inducible plasmid containing BlpA1 + BlpA2 + BltA on xylose-containing media. Serial dilutions are shown in the representative image on the left and results from three independent experiments are quantified in the panel on the right. Data in panels B and C represent the mean of three independent experiments and error bars represent standard deviation and comparisons in which *P* < 0.05 are displayed. Statistics in panel B reflect the ordinary one-way ANOVA with Dunnett correction for multiple comparisons and statistics in panel **C** reflect the student’s *t*-test.

As T7SS-associated LXG proteins have been shown to have toxic activity to other bacteria, we sought to investigate whether BltA might be broadly toxic upon intracellular expression. To evaluate this, we cloned *bltA* into the pSCRhaB2 vector under the control of a rhamnose inducible promoter as performed previously by others (8, 12–14). Induction of the full-length BltA or the C-terminal BltA toxin domain alone in liquid culture had no or little impact on *E. coli* growth, respectively (Fig. 4A; Fig. S5A). To assess whether chaperones are required for BltA toxicity, we co-expressed *bltA* along with *blpA1*, *blpA2*, or both *blpA1 and blpA2* together. Compared to *E. coli* harboring an empty vector control, we observed a drastic reduction of growth in the liquid culture of *E. coli* expressing *blpA1* and *blpA2* with *bltA*, but not upon expression of *blpA1-2* alone (Fig. 4A). Interestingly, we found that co-expression of either *blpA1* or *blpA2* with *bltA* was sufficient for this inhibition of *E. coli* growth (Fig. 4A) but that none of these conditions appear to result in cell lysis (as measured by OD_600_). To further investigate the impact of BltA on *E. coli* viability, we plated serial dilutions of the above strains on solid media containing or lacking the rhamnose inducer. Similar to the above growth assays, induction of expression of either *bltA* or *blpA1-2* alone did not impact *E. coli* CFU recovery. Interestingly, we found that co-expression of either *blpA1* or *blpA2* with *bltA* significantly decreased *E. coli* viability compared to the empty vector control, whereas co-expression of both *blpA1-2* with *bltA* only impacted *E. coli* colony morphology (Fig. 4B). As strains plated on solid agar lacking rhamnose (uninduced) exhibited equivalent CFU recovery (Fig. S5B)**,** these data suggest that intracellular induction of BltA along with a chaperone is toxic to *E. coli*.

To investigate whether BltA is also toxic when expressed within Gram-positive bacteria, we next assessed BltA inhibition of *S. aureus,* which was also not susceptible to GBS T7SS in direct competition experiments (Fig. 1A). To evaluate this, we cloned *blpA1-2* and *bltA* into the pEPSA5 expression vector under the control of a xylose-inducible promoter and plated serial dilutions on solid media containing or lacking the xylose inducer. We found that induction of *blpA1-2* and *bltA* intracellular expression resulted in approximately 1,000-fold decreased CFU recovery of *S. aureus* compared to the empty vector (Fig. 4C) and uninduced controls (Fig. S5C). These data suggest that BltA is capable of intoxicating both Gram-negative and Gram-positive hosts intracellularly and that its target is likely conserved across genera.

### BltA toxicity is abrogated by a missense mutation within the C-terminal putative toxin domain

**Fig 5:**
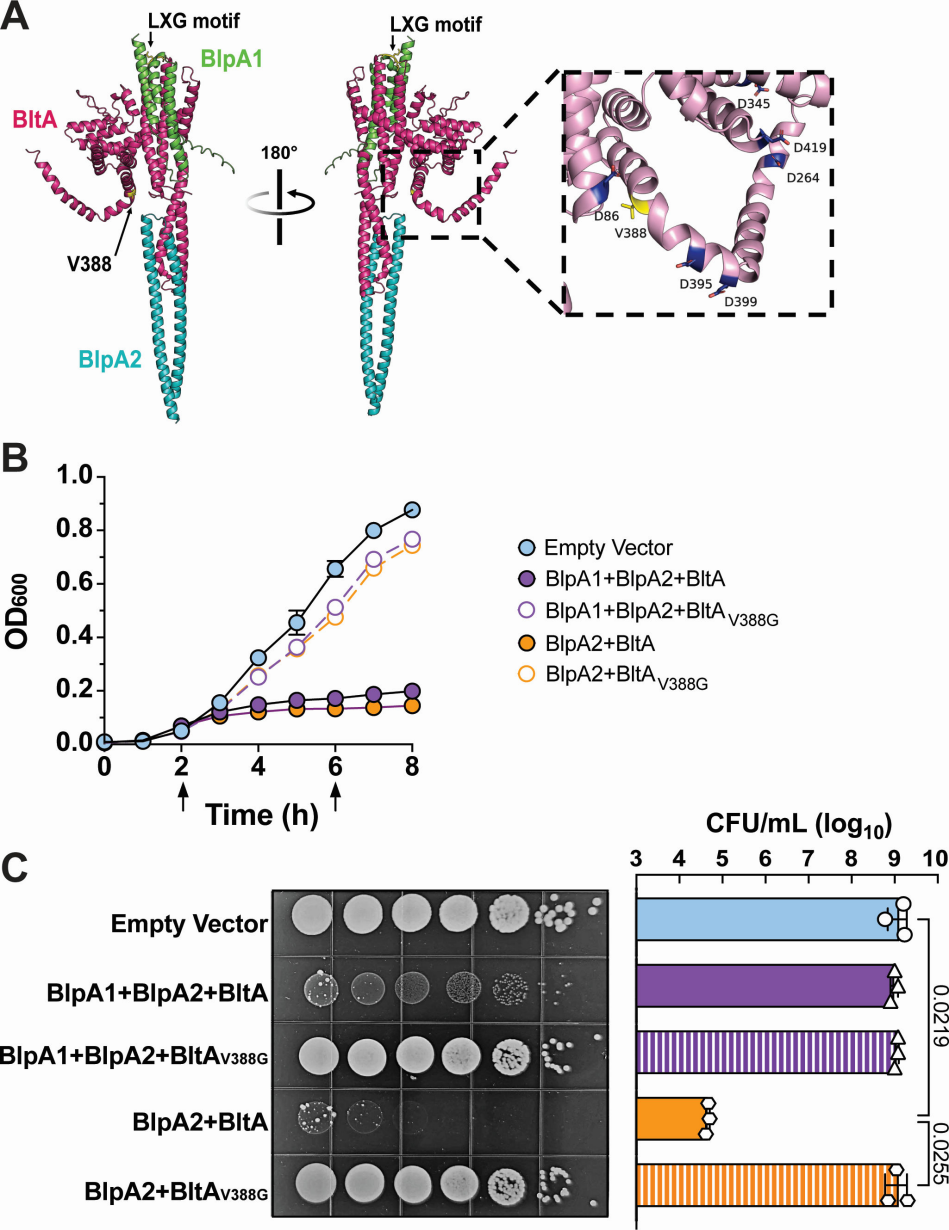
Identification of BltA-resistant *E. coli*. BltA-resistant colonies identified in Fig. 4 were isolated and their plasmids were sequenced to identify mutations abrogating toxicity. (A) AlphaFold Multimer predicted model of BltA, BlpA1, and BlpA2 labeled in magenta, green, and blue, respectively. The N-terminal LXG motif and residue V388 are highlighted in yellow. Modeling confidence metrics are presented in Fig. S6. Growth of *E. coli* in (B) liquid or on (C) solid medium is not impacted by induction of BltA harboring the V388G mutation compared to the empty vector control. CFU recovery of uninduced strains from panel C is provided in Fig. S7B. Data in panels B and C represent the mean of three independent experiments and error bars represent the standard error of the mean and standard deviation, respectively. Statistics in panel C reflect the ordinary one-way ANOVA with Tukey’s multiple comparison test and comparisons in which *P* < 0.05 are displayed.

In performing the above inducible intoxication assays, *bltA* expression along with either *blpA1* or *blpA2* resulted in a dramatic reduction in *E. coli* viability. Interestingly, in these assays, we often observed resistant colonies that were unaffected by *bltA* induction (Fig. 4B). We isolated single resistant colonies and sequenced their plasmids to identify potential mutations that may impact BltA’s toxic activity. We found many mutations across these plasmids, including large insertions within *bltA* or its associated chaperones, a slip-strand mutation within *bltA* resulting in a frameshift, as well as a missense V388G mutation within the putative *bltA* toxin domain (Fig. S7A; Fig. 5A). Induction of *bltA*_V388G_ co-expressed with *blpA2* within a clean *E. coli* background (to exclude any additional chromosomal mutations) significantly reduced BltA inhibition in both liquid and agar media (Fig. 5B and C). Furthermore, *bltA*_V388G_ co-expressed with both *blpA1-2* reduced BltA growth inhibition in liquid agar and restored the altered *E. coli* morphology observed in cells expressing *blpA1-2* and *bltA* (Fig. 5B and C). These data suggest that this V388 residue may impact BltA’s function.

### BltA and BlpA1-2 chaperones promote GBS vaginal colonization

**Fig 6:**
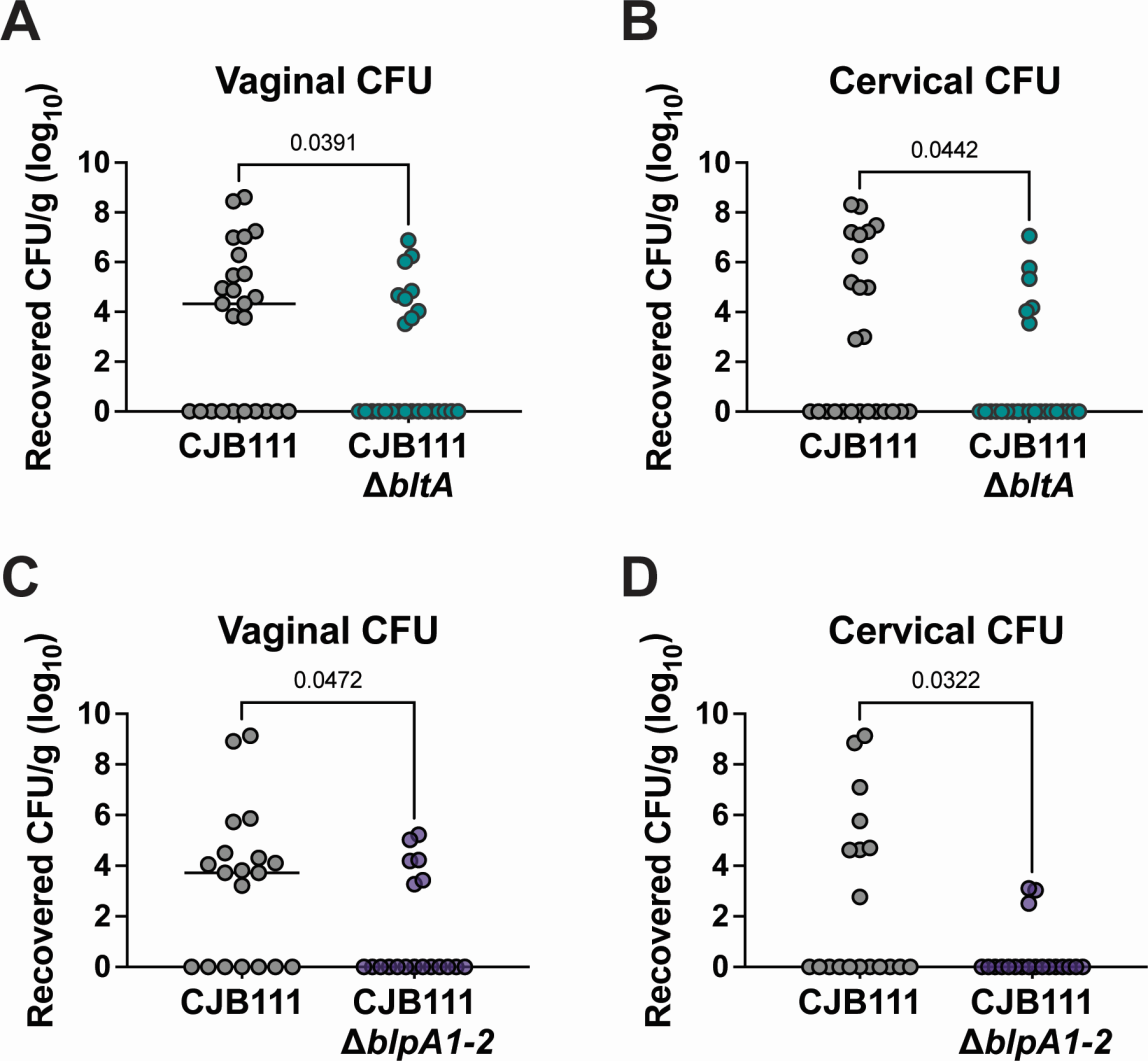
Loss of BltA or associated chaperones impacts GBS vaginal colonization by T7SS subtype I strain CJB111. Recovered CFU counts from the (A, C) vaginal and (B, D) cervical tissue of mice mono-colonized with parental CJB111 GBS and either the CJB111∆*bltA* or CJB111∆*blpA1-2* mutant strains. Each dot represents one mouse and all independent experiments’ data are combined in these figures (*n* = 25/group for Δ*bltA* experiments and *n* = 19/group for Δ*blpA1-2* experiments). The bars in these plots show the median and statistics represent the Mann-Whitney U test.

Previously, we showed that the T7SS is important for colonization of the vaginal tract by subtype I-expressing strain CJB111. However, the T7SS effectors that mediate this colonization were not investigated. Here we sought to determine whether BltA might impact GBS colonization. To investigate this, we performed single-challenge vaginal colonization experiments inoculating either parental CJB111 or the isogenic CJB111∆*bltA* mutant into the vaginal tract and assessed GBS persistence in female genital tract tissues. Similar to those observed with our T7SS-deficient CJB111∆*essC* mutant (24), we observed differences in tissue burdens in the vagina and cervix between mice colonized with the CJB111∆*bltA* mutant compared to parental CJB111 (Fig. 6A and B). As the BlpA1-2 chaperones appear to be important for BltA toxicity, we further hypothesized that the loss of these chaperones might also impact GBS persistence within female genital tract tissues. Indeed, upon GBS single challenge into the vaginal tract, we observed that the CJB111∆*blpA1-2* mutant was recovered in significantly lower burdens from the vagina and cervix compared to parental CJB111 (Fig. 6C and D). These results further demonstrate the importance of BltA and its associated chaperones during *in vivo* GBS colonization.

## DISCUSSION

Herein, we demonstrate that GBS T7SS reduces recovery of mucosal pathobiont *E. faecalis*, both *in vitro* and *in vivo* within the complex environment of the murine female genital tract. We have further identified BltA as an LXG toxin encoded downstream of T7SS machinery in GBS subtype I that contributes to this competition and that is toxic to other bacteria upon intracellular expression. Investigation of resistant *E. coli* revealed mutations within the chaperones and BltA, including a BltA V388G missense mutation that abrogates toxicity. Finally, we show that BltA and its associated chaperones promote GBS persistence within the vagina and cervix during colonization.

In this work, we primarily examined GBS in competition with other pathobionts such as *Enterococcus faecalis*, which is another vaginal opportunistic pathogen (31) that may concurrently colonize the female genital tract during conditions such as aerobic vaginitis (37, 38). We observed T7SS-mediated *E. faecalis* antagonism by two different GBS clinical isolates and reciprocally found that CJB111 GBS T7SS (subtype I) is capable of inhibiting a panel of *E. faecalis* isolates, although we observed a range of inhibition. Our future work will investigate *E. faecalis* determinants of GBS T7SS susceptibility. Interestingly, under the conditions used here, we did not observe GBS T7SS subtype I-mediated competition with human vaginal commensal *Lactobacillus crispatus* and instead observed a T7SS-independent decrease in GBS CFU recovery following co-culture. Consistently, lactobacilli have been shown previously to impact GBS virulence and viability and have therefore been suggested as a potential therapeutic to reduce or prevent GBS colonization of the vaginal mucosa (21, 39, 40). While future work will investigate the interaction of the GBS T7SS with other vaginal commensals, it is possible that the T7SS may have evolved to target other pathobionts to promote niche establishment rather than members of the native vaginal microbiota; however, this remains to be determined. Importantly, while T7SSb-mediated interbacterial antagonism has been demonstrated for several Gram-positive T7SSb^+^ organisms, the conditions used during predator-prey assays likely influence whether the T7SSb is induced and therefore whether LXG toxin-mediated competition is observed or not (27, 41). Therefore, identifying factors and conditions that induce GBS T7SSb expression is part of our ongoing work.

We further show here that induction of intracellular BltA expression is toxic to both *S. aureus* and *E. coli*. While no obvious conserved domain or motifs exist within the BltA C-terminal toxin domain to inform its function, based on the above data, the target of this toxin appears to be intracellular and conserved across Gram-positive and Gram-negative bacteria. Potentially corroborating this, many BltA resistance-conferring mutations identified during *E. coli* intoxication assays were IS-mediated and, in T5SS, it was observed that increased IS transposition was more associated with DNase toxins relative to toxins with other intracellular targets (42). Determining the function of BltA comprises a direction of our future work. Because neither *S. aureus* nor *E. coli* was inhibited by GBS during predator-prey assays, it is possible that an extracellular factor may be required for T7SS-mediated antagonism. To date, it is unclear how T7SSb effectors, including LXG toxins, are delivered into a prey cell, although a few studies have indicated that T7SSb competition is contact-dependent (11, 13, 26). We hypothesize that specific extracellular determinants may dictate the range of GBS T7SS antagonism and will investigate this in our future work.

During inducible intoxication assays in *E. coli*, we found that BltA alone was not sufficient to reduce bacterial viability but that co-expression of BlpA1 and/or BlpA2 promoted BltA-mediated inhibition in liquid medium. Furthermore, some of the mutations we identified in resistant colonies disrupted the BlpA putative chaperones or the N-terminal LXG domain of BltA that is predicted to interact with these chaperones. This is consistent with previous literature that Laps may promote LXG stability. Interestingly, co-expression of a single chaperone with BltA resulted in decreased *E. coli* viability during solid agar growth assays compared to expression of BltA with both BlpA1 and BlpA2, indicating that specific chaperone interactions may impact BltA toxicity. As we observe chaperone-dependent BltA toxicity, we hypothesize that chaperones may be required for BltA antagonism of other bacteria. However, whether BlpA1-2 is co-secreted with BltA and delivered as a complex to a prey cell needs to be assessed experimentally.

In addition to “Lap” chaperones, a recent study showed that a DUF4176 protein, often encoded within T7SS loci, is also required for LXG toxin secretion in *S. intermedius* (33). In *S. aureus,* a DUF4176 protein was further found to directly interact with the LXG toxin TspA, along with its corresponding Lap proteins, and to contribute to TspA secretion (14). We previously identified genes encoding for DUF4176 proteins downstream of *bltA* (24)*,* but their role has yet to be investigated in GBS. Given the above-known associations between DUF4176 proteins and LXG toxins, we hypothesize that the GBS DUF4176 protein encoded for downstream of *bltA* may interact with and participate in the secretion of BltA. Future work will investigate molecular interactions of BlpA1, BlpA2, and the DUF4176 protein with BltA well as the role of these putative chaperones in secretion, stability, and/or toxin function.

Upon further sequencing of resistant colonies, we also identified a V388G mutation within the BltA putative toxin domain in two independent experiments. As the co-expression of BltA_V388G_ with its chaperone(s) is not toxic to *E. coli*, it is possible that the V388G mutation may disrupt the stability and/or function of BltA. Although this region of the model is predicted by AlphaFold at very low confidence, this V388G mutation occurs within the C-terminal putative toxin domain of BltA and is proximal to several aspartate residues in multiple predictive iterations (Fig. 5A). Aspartate-rich regions are associated with some enzyme active sites and an aspartate-rich motif has been identified previously within the *Streptococcus intermedius* LXG toxin TelC to be important for its function (13). Whether BltA functions as an enzyme is still unknown and our future work will investigate whether these aspartate residues are important for BltA toxicity and how mutations in this area impact the structure and function of BltA.

While in this study we have focused on T7SSb effectors encoded by the highly prevalent GBS T7SS subtype I, other subtypes of GBS T7SS remain unstudied. GBS T7SS subtypes I–III encode similar types of putative effectors and accessory proteins, including LXG toxins, associated chaperones, transmembrane (Tm) proteins, and DUF4176-containing proteins. Despite this conservation, these protein products are largely unique between subtypes at the sequence level. Indeed, while each putative LXG toxin from subtypes I-III contains the conserved N-terminal LXG domain, each encodes a unique C-terminal putative toxin domain. We have observed previously that many of these LXG toxins lack easily identifiable functional domains; thus, the biochemical activity of these proteins must be determined experimentally. Here, we observed that the subtype I LXG toxin BltA contributes to *E. faecalis* antagonism. As we also observed T7SS subtype III-dependent *E. faecalis* inhibition by clinical isolate CNCTC 10/84, future work will investigate the potential contribution of the subtype III LXG protein to competition as well as the potential antagonistic range of the additional GBS T7SS subtypes II and IV.

Toxins across many specialized secretion systems commonly encode an immunity factor nearby to prevent self-intoxication. Indeed, in other bacterial species in which LXG toxins have been studied, a cognate immunity factor was identified in either one or two genes downstream of the toxin-encoding gene. Within the GBS subtype I locus, *bltA* is followed by two genes of unknown function. To determine whether the Tm protein-encoding gene, *ID870_4220*, encoded immediately downstream of *bltA* might encode for the BltA immunity protein we expressed *ID870_4220* in *E. faecalis* in predator-prey assays. Interestingly, we did not observe 4220-mediated abrogation of T7SS-dependent competition in the conditions tested (Fig. S3B). To further validate this, we next scanned other bacterial genomes for BltA homologs to evaluate whether a common gene is always encoded downstream, which would strongly indicate its function as an immunity factor. Upon BLAST search of BltA in non-GBS genomes, we identified a homolog (83% identical) in the mastitis pathogen *Streptococcus uberis* (GenBank accession SAMN27553288). This *S. uberis* locus encodes an LXG module syntenic to that found in the subtype I GBS (Fig. S8), including homologs of BlpA1 (72% identical) and BlpA2 (41% identical) encoded for upstream of BltA. The *S. uberis* locus downstream of *bltA* harbors genes encoding for a Tm domain-containing protein as well as a DUF4176 domain-containing protein (85% identical), similar to those found in GBS subtype I. Interestingly, the *S. uberis* Tm-encoding gene contains two slip-strand mutations resulting in a frameshift (putatively resulting in the correct translation of 115 of 182 amino acids). Similar to EssC and EsaA truncation observed in our previous work (24), NCBI ORF Finder predicts that the remainder of this Tm protein may be encoded as a second ORF, but it remains to be seen how these mutations impact the *S. uberis* T7SS. Based on this and our above results, the 4220 Tm protein may not be sufficient to abrogate BltA toxicity. However, it is possible that overexpression of the 4220 Tm protein in competition assays may result in mis-localization, yielding a false-negative result under the experimental conditions tested or, in the above case of *S. uberis*, that the truncated Tm protein is sufficient to prevent self-intoxication. Alternatively, BltA may be tightly regulated so as to not require an endogenous downstream immunity factor under laboratory conditions or another downstream gene may encode for the BltA immunity factor. This comprises a direction in our future work.

To conclude, in this work, we have demonstrated GBS T7SS-dependent inhibition of *E. faecalis in vitro* for two distinct T7SS subtypes as well as T7SS subtype I-dependent *E. faecalis* antagonism in the murine female genital tract *in vivo*. We identified a novel GBS T7SS LXG effector, BltA, as a mediator of this interbacterial antagonism. Our work further indicates a role for BltA as an antibacterial toxin with an intracellular target conserved across Gram-positive and Gram-negative genera. As has been seen in multiple streptococcal species as well as *S. aureus*, we observed that two putative ɑ-helical chaperone proteins, BlpA1 and BlpA2, are important for BltA toxicity. Future work will characterize the mechanisms driving BltA toxicity including the function of BlpA1 and BlpA2, the target of BltA, and identifying the cognate immunity factor. In addition, we seek to identify prey bacterial factors that contribute to the breadth and specificity of GBS T7SS-mediated interbacterial antagonism against not only vaginal pathobionts but also those found in broader GBS host polymicrobial niches such as the neonatal gastrointestinal tract as well as in diabetic wounds.

## MATERIALS AND METHODS

### Bacterial strains

In this study, we primarily use a GBS isolate background that encodes a subtype I T7SS, CJB111 (43). Other bacterial strains and mutants used in this study can be found in Table S1. GBS strains were grown statically in Todd Hewitt broth (THB; Research Products International, RPI Catalog# T47500) at 37°C with erythromycin (5 µg/mL) when applicable. *E. coli* strains were grown shaking in Luria broth (LB; RPI Catalog# L24066) at 37°C supplemented with erythromycin (500 µg/mL), carbenicillin (100 µg/mL), chloramphenicol (15 µg/mL), trimethoprim (200 µg/mL), or L-rhamnose (0.1% wt/vol) when applicable. *S. aureus* strains were grown shaking in Tryptic soy broth (TSB; Becton Dickinson, BD Catalog# 211822) at 37°C supplemented with chloramphenicol (10 µg/mL) or xylose (2% wt/vol) when applicable. *E. faecalis* strains were grown shaking in THB at 37°C with chloramphenicol (15 µg/mL) when applicable. *S. epidermidis* was grown shaking in TSB at 37°C. *S. pyogenes* was grown statically in THB at 37°C in 5% CO_2_. *L. crispatus* was grown statically in de Man, Rogosa, Sharpe broth (MRS; ThermoFisher Scientific Catalog #CM0361) at 37°C in 5% CO_2_.

### Cloning

Clean *bltA and blpA1-2* deletion mutants were created *via* allelic exchange in CJB111 using the temperature-sensitive plasmid pHY304 and using a gene encoding spectinomycin resistance (*aad9*) in the knockout construct. Second crossover mutants were screened for erythromycin sensitivity and spectinomycin resistance. For gene expression in *E. faecalis, ID870_4220* was amplified from CJB111 using Phusion polymerase and ligated into the constitutive *E. faecalis* expression vector pLZ12A *via* Gibson assembly. For intoxication assays, *blpA1, blpA2,* and/or *bltA* were amplified from the CJB111 genome using Phusion polymerase and ligated into the inducible *E. coli* expression vector pSCRhaB2 or the inducible *S. aureus* expression vector pEPSA5 *via* Gibson assembly. Constructs for the Nanobit nanoluciferase assay were generated by adding a pep86 tag and short linker sequence (provided by Promega) to the 3′ end of *bltA* using Phusion polymerase. Amplicons were then ligated into the GBS expression vector pDCErm using Quick Ligase (restriction enzymes BamHI-HF and SacII-HF) or Gibson assembly. Strains created and primers used in this study can be found in Table S1.

### Interbacterial competition

GBS predator and designated prey strains were grown overnight for 16–20 hours under the standard laboratory conditions listed above for each strain. Except for *L. crispatus,* all prey co-cultures were prepared as follows: for bacteria that were not grown overnight in THB, cells were first pelleted, the supernatant discarded, and finally resuspended in fresh THB. All strains were then normalized to OD_600_ = 0.4 in THB. Prey strains were diluted 1:1,000 in THB. 10-fold serial dilutions of each prepared predator and prey culture were plated on respective standard agar media and incubated under respective standard laboratory conditions to confirm predator-prey CFU ratios. Competition cultures were prepared by mixing 1 mL of normalized GBS predator culture with 1 mL of prepared prey culture in a 5 mL culture tube (Falcon, Catalog# 352054). Cultures were then vortexed briefly before being incubated statically at 37°C, 5% CO_2_ for 24 hours. Monoculture controls were included wherein each prepared culture was mixed with 1 mL THB rather than a second strain. After 24 hours, serial dilutions of each co-culture were plated on differential and/or selective media to assess the final CFU of both GBS and the designated prey strain. Medias used were as follows: CHROMagar StrepB (CHROMagar Catalog# SB282) for GBS; THA containing rifampin (50 µg/mL) and fusidic acid (25 µg/mL) for *E. faecalis* OG1RF and CHROMagar StrepB for all other *E. faecalis* strains*;* mannitol salt agar (BD, Catalog# 211407) for *S. aureus* and *S. epidermidis*; THA and THA containing tetracycline (5 µg/mL) for *S. pyogenes* (CFU recovered from THA with tetracycline were subtracted from CFU recovered from THA to calculate *S. pyogenes* CFU); and MacConkey agar (BD Catalog# 212123) for *E. coli*. Monocultures were plated on the same media as the input cultures. All plates were then incubated 16–24 hours at 37°C (with 5% CO_2_ for *S. pyogenes*). CFU was enumerated for each strain and prey relative viability was calculated as a ratio of the prey CFU recovered from co-culture with parental GBS compared to co-culture with mutant GBS. This competition protocol was slightly modified for *L. crispatus* competition due to inherent lactobacilli growth constraints. Rather than normalizing and incubating strains in THB, both GBS and *L. crispatus* were prepared and incubated in MRS. Control experiments were performed to confirm there was no growth defect of GBS in MRS (data not shown). Monocultures and co-cultures were set up as described above. *L. crispatus* monocultures and co-cultures were plated on MRS agar and incubated in 5% CO_2_. *L. crispatus* CFU was enumerated, and relative viability was then calculated as above.

### Vaginal colonization

GBS vaginal colonization was assessed using a previously described murine model of vaginal persistence and ascending infection (44). Briefly, 8- to 10-week-old female CD1 (Charles River) mice were synced with beta-estradiol at day −1 and inoculated intravaginally with mid-log grown GBS (approximately 1 × 10^7^ CFU) in PBS on day 0. Post-inoculation, mice were lavaged with PBS daily, and the samples were serially diluted and plated for CFU counts to determine bacterial persistence on CHROMagar. At experimental endpoints (upon vaginal clearance by 70% of the mice of either group), mice were euthanized, and female genital tract tissues (vagina and cervix) were collected. Tissues were homogenized and samples were serially diluted and plated on CHROMagar for CFU enumeration. Bacterial counts were normalized to the tissue weight. For experiments using the *bltA* mutant, 1 mouse of 26 in each group was excluded on the basis of not being initially colonized. For experiments using the *blpA1-2* mutant, 1 mouse of 20 in each group was excluded on the basis of having cleared for 2 lavage points (across 4 days) but then were potentially re-infected as on the last day we recovered GBS levels higher than those on Day 1.

Co-challenge GBS-*E. faecalis* vaginal colonization was performed as described above, except that mice were co-challenged with approximately 5 × 10^6^ CFU *E. faecalis* + 5 × 10^6^ CFU GBS. Lavage and tissues were serially diluted and plated on CHROMagar for GBS enumeration and THA containing rifampin (50 µg/mL) and fusidic acid (25 µg/mL) for *E. faecalis* enumeration. Co-colonization experiments were ended upon vaginal clearance of *E. faecalis* by 70% of either group of mice. These experiments were approved by the committee on the use and care of animals at the University of Colorado-Anschutz Medical Campus (protocol #00316).

### Detection of cell-associated and secreted T7SS effectors by western blot

Secretion of EsxA and BltA during laboratory growth was detected as described previously (7), with slight modifications for strains expressing pep86 tagged effectors. Briefly, GBS strains harboring pep86-tagged constructs and respective empty vector strains were grown with the addition of erythromycin (5 µg/mL) to the culture media. Stationary phase GBS cultures were separated into pellet and supernatant fractions *via* centrifugation. Supernatants were filtered (Millex Low Protein Binding Durapore PVDF Membrane 0.22 µm filters, catalog #SLGVR33RS), supplemented with an EDTA-free protease inhibitor cocktail (Millipore-Sigma set III, catalog # 539134; 1:250 dilution), and proteins were precipitated with trichloroacetic acid [resuspended in Tris buffer (50 mM Tris HCl, 10% glycerol, 500 mM NaCl, pH 7)]. Bacterial pellets were lysed by bead-beating in Tris buffer + protease inhibitor and supplemented with Triton-X-100 to solubilize membrane proteins. Supernatant and pellet fractions were run on SDS-PAGE and stained with Coomassie stain or transferred to membranes as described previously. Membranes were blocked in LI-COR blocking buffer and then probed with anti-HiBiT (pep86) mouse monoclonal antibody (1 µg/mL; Promega, Catalog# N7200) and/or anti-EsxA1 rabbit polyclonal antibody (0.5 µg/mL; GenScript) in LI-COR blocking buffer overnight at 4°C. Following washes, membranes were incubated with IRDye 800CW goat anti-mouse IgG and/or IRDye 680RD goat anti-rabbit IgG secondary antibodies from LI-COR (1:10,000 dilution; 1 hour, room temperature). Following washes in TBST and water, western blots were imaged using the LI-COR Odyssey.

### Detection of cell-associated and secreted T7SS effectors by Nanobit assay

GBS strains harboring pep86-tagged constructs and respective empty vector strains were grown as in the western blot protocol above, with the addition of erythromycin (5 µg/mL) to the culture media. Pellet and supernatant fractions were prepared as above. Pellet and concentrated supernatant samples were diluted 1:2 or 1:10, respectively, in Tris buffer. Then, 100 µL of each sample was added to a 96-well white microplate (Costar). The pep86-tag was detected in each fraction using the Nano-Glo HiBiT Extracellular Detection System (Promega Catalog# N2420) according to the manufacturer’s instructions. Luminescence was measured with a Tecan Infinite M Plex plate reader. Relative luminescence from BltA-pep86 expressing parental CJB111 or CJB111∆*essC* strains was normalized to their respective empty vector controls.

### Bacterial intoxication assays

*E. coli* strains containing pSCRhaB2 constructs were grown shaking overnight in LB with trimethoprim (200 µg/mL) at 37°C. Each strain was then normalized to OD_600_ = 1 before being diluted 1:100 into fresh LB with trimethoprim (200 µg/mL). For OD_600_ growth curves, 150 µL of each strain was added to a 96-well microtiter plate in technical triplicate. Plates were grown shaking at 800 rpm, 37°C and OD_600_ was measured every hour with a Tecan Infinite M Plex plate reader. Genes expressed on pSCRhaB2 were induced at 2 and 6 hours with 0.1% wt/vol L-rhamnose (or at 2, 4, 6, and 8 hours for the C-terminal BltA construct). Uninduced controls were included for each experiment. For agar toxicity assays, OD_600_ normalized cultures from above were serially diluted 10-fold to 10^−8^ in PBS and 10 µL of each dilution was spotted onto LB agar plates containing trimethoprim (200 µg/mL) with or without 0.1% wt/vol L-rhamnose. Plates were then incubated for 16 hours at 37°C before imaging and enumerating CFU. *S. aureus* pEPSA5 strains were grown shaking overnight in TSB with chloramphenicol (10 µg/mL) at 37°C. Each strain was then normalized to OD_600_ = 0.5 before being serially diluted 10-fold to 10^−8^ in PBS. 10 µL of each dilution was spotted onto TSA containing chloramphenicol (10 µg/mL) with or without 2% wt/vol xylose. Plates were incubated for 16 hours at 37°C before imaging and enumerating CFU.

### AlphaFold modeling

Predictive modeling of the BlpA1-BlpA2-BltA complex was performed using AlphaFold2-multimer weights (45) and implemented in ColabFold (46). Illustrations were generated using PyMOL (The PyMOL Molecular Graphics System, Version 2.5.5, Schrödinger, LLC) (47).

### Statistics

Statistical analysis was performed using Prism version 10.2.3 for macOS (GraphPad Software, La Jolla, CA, United States). Significance was defined as *P* < α, with α = 0.05.

## Data Availability

All relevant data are within the manuscript and its Supporting Information files.

## SUPPLEMENTAL MATERIAL

Supplemental figures mbio.02088-24-s0001.pdf Figures S1 to S8.

Table S1 mbio.02088-24-s0002.xlsx Strains and primers used in this study.

ASM does not own the copyrights to Supplemental Material that may be linked to, or accessed through, an article. The authors have granted ASM a non-exclusive, world-wide license to publish the Supplemental Material files. Please contact the corresponding author directly for reuse.
