# Supplementary material for: A group B streptococcal type VII-secreted LXG toxin mediates interbacterial competition and colonization of the murine female genital tract
Source: mBio. 2024 Aug 27;15(10):e02088-24. doi: 10.1128/mbio.02088-24 (PMC11481500; doi:10.1128/mbio.02088-24)
Supplement: Supplemental figures — Figures S1 to S8. [file mbio.02088-24-s0001.pdf]

SUPPLEMENTARY INFORMATION

A group B streptococcal type VII secreted LXG toxin mediates interbacterial competition and colonization of the murine female genital tract

Alyx M. Job[1], Kelly S. Doran*[1], Brady L Spencer*[1]

[1]University of Colorado-Anschutz, Department of Immunology and Microbiology, Aurora, CO, USA

***Co-corresponding authors**:
Brady L. Spencer and Kelly S. Doran
Department of Immunology and Microbiology
University of Colorado-Anschutz
Email: brady.spencer@cuanchutz.edu, kelly.doran@cuanschutz.edu

Running title: GBS T7SS promotes interbacterial competition

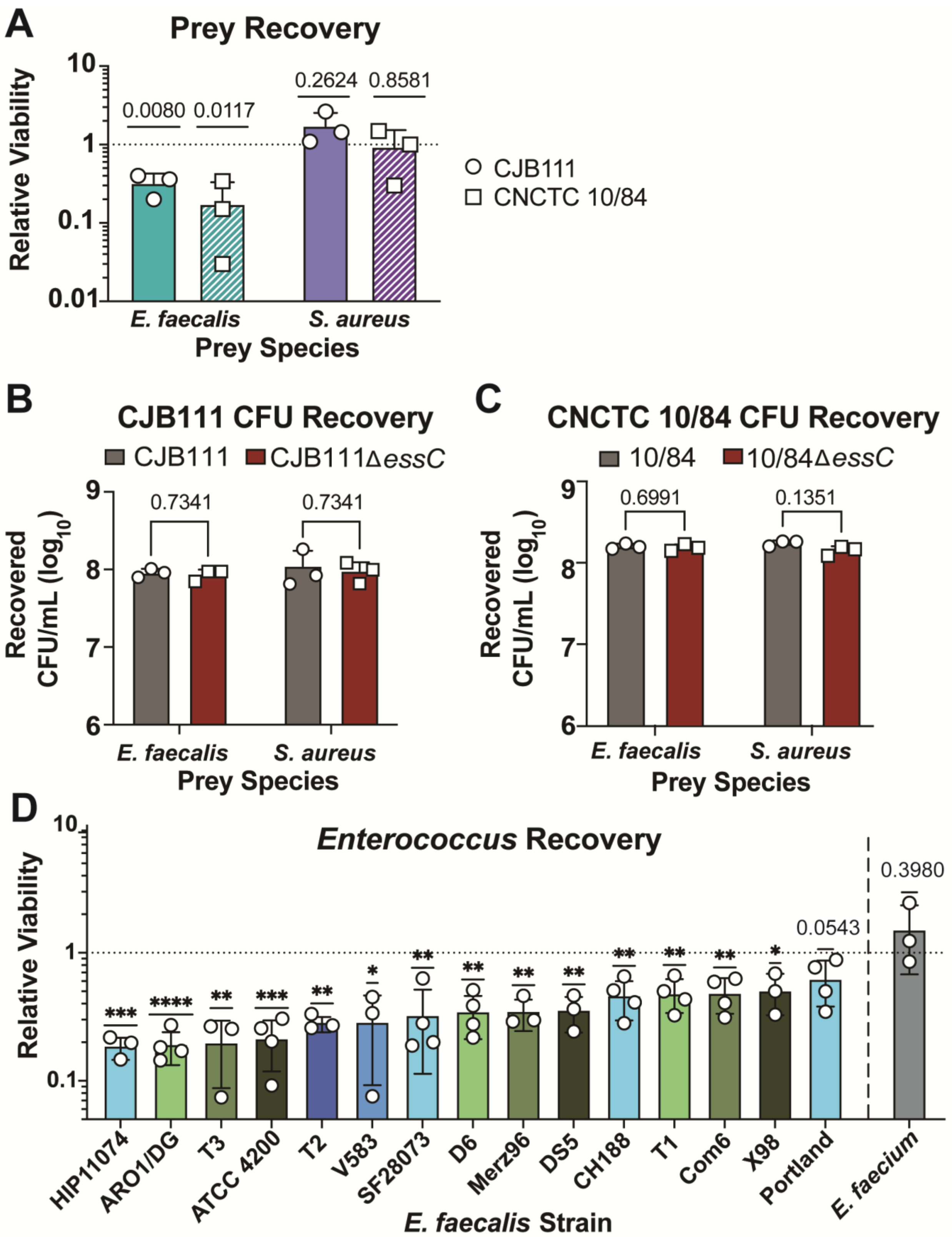

A
Prey Recovery
Relative Viability
10
1
0.1
0.01
0.0080
0.0117
0.2624
0.8581
CJB111
CNCTC 10/84
E. faecalis
S. aureus
Prey Species
B
CJB111 CFU Recovery
CJB111
CJB111ΔessC
Recovered CFU/mL (log10)
9
8
7
6
0.7341
0.7341
E. faecalis
S. aureus
Prey Species
C
CNCTC 10/84 CFU Recovery
10/84
10/84ΔessC
Recovered CFU/mL (log10)
0.6991
0.1351
E. faecalis
S. aureus
Prey Species
D
Enterococcus Recovery
Relative Viability
0.3980
0.0543
***
****
**
***
**
*
**
**
**
**
**
**
**
*
HIP11074
ARO1/DG
T3
ATCC 4200
T2
V583
SF28073
D6
Merz96
DS5
CH188
T1
Com6
X98
Portland
E. faecium
E. faecalis Strain

SFig. 1. GBS isolate CNCTC 10/84 displays a similar interbacterial antagonism profile to CJB111. A) Predator-prey interbacterial competition assay performed between parental or Δ*essC* mutants of GBS CJB111 or CNCTC 10/84 and prey *E. faecalis* or *S. aureus.* Prey relative viability is calculated as in **Fig. 1A**. Statistics reflect one-sample t-tests against a hypothetical value of 1. Data represent the mean of three independent experiments and error bars represent standard deviation. Parental or Δ*essC* mutant GBS CFU recovery for **B)** CJB111 and **C)** CNCTC 10/84 strains following *in vitro* predator-prey inter-species competition experiments performed in **SFig. 1A**. Statistics reflect multiple unpaired t-tests. Data represent the mean of three independent experiments and error bars represent standard deviation. **D)** Predator-prey interbacterial competition assay performed between parental CJB111 or Δ*essC* mutants and a panel of 15 prey *E. faecalis* isolates. Prey relative viability is calculated as in **Fig. 1A**. Statistics reflect one-sample t-tests against a hypothetical value of 1. Data represent the mean of at least three independent experiments and error bars represent standard deviation. $p < 0.05$, *; $p < 0.01$, **; $p < 0.001$,

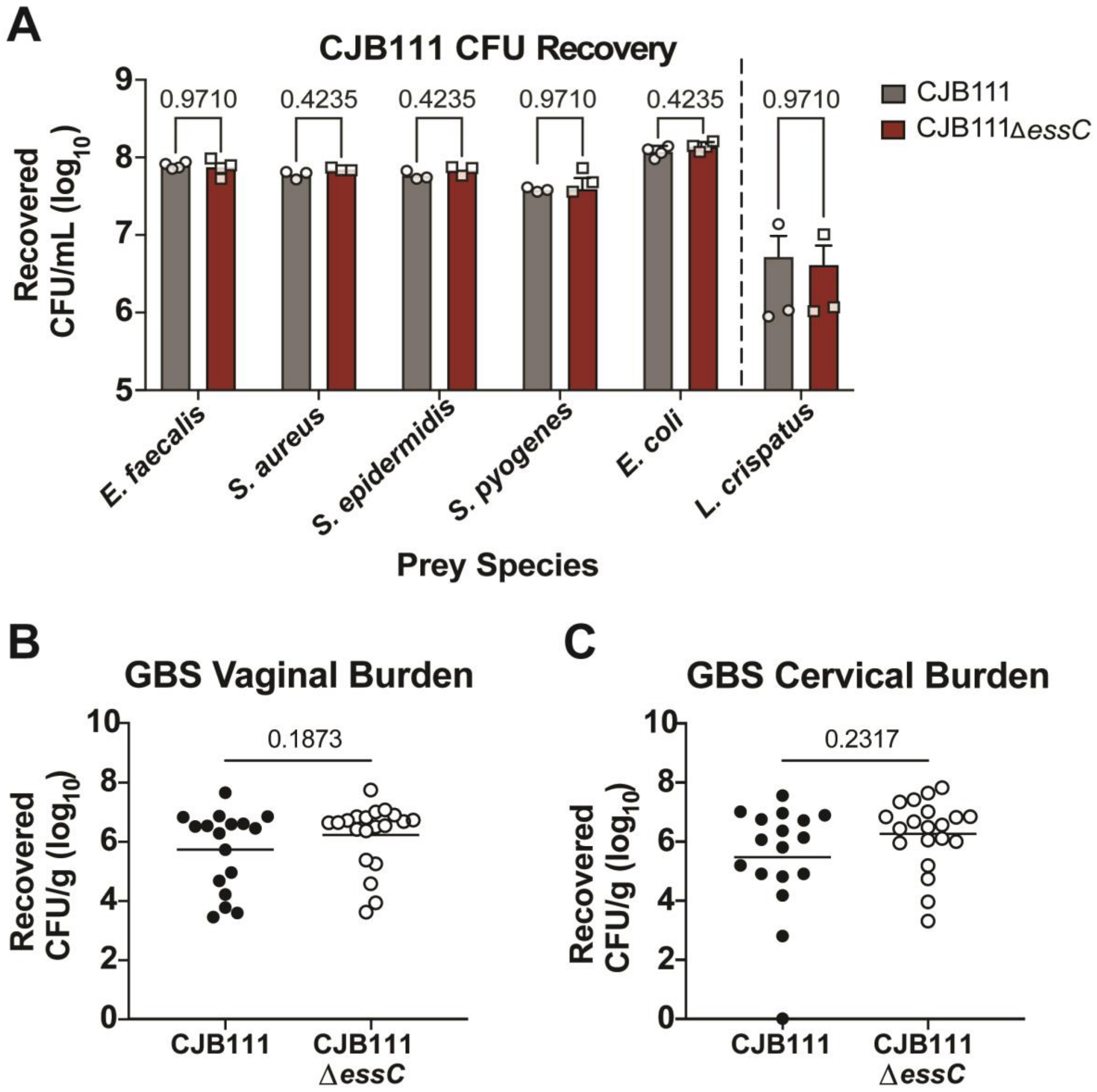

SFig. 2. Parental CJB111 and CJB111Δ*essC* CFU are recovered equally upon co-culture with a given prey during *in vitro* and *in vivo* competition assays. A) Parental CJB111 and CJB111Δ*essC* mutant CFU recovery following *in vitro* predator-prey inter-species competition experiments performed in **Fig. 1A**. Statistics reflect multiple unpaired t-tests. Data represent the mean of three independent experiments and error bars represent standard deviation. GBS CFU burden recovered from the **B)** vagina and **C)** cervix during *in vivo* co-colonization competition experiments performed in **Fig. 1C-D**. Each dot represents one mouse and data from two independent experiments are combined in these figures (n = 17, 20 total in parental and Δ*essC* mutant co-colonization groups, respectively). The bars in these plots show the median and statistics represent the Mann Whitney U test.

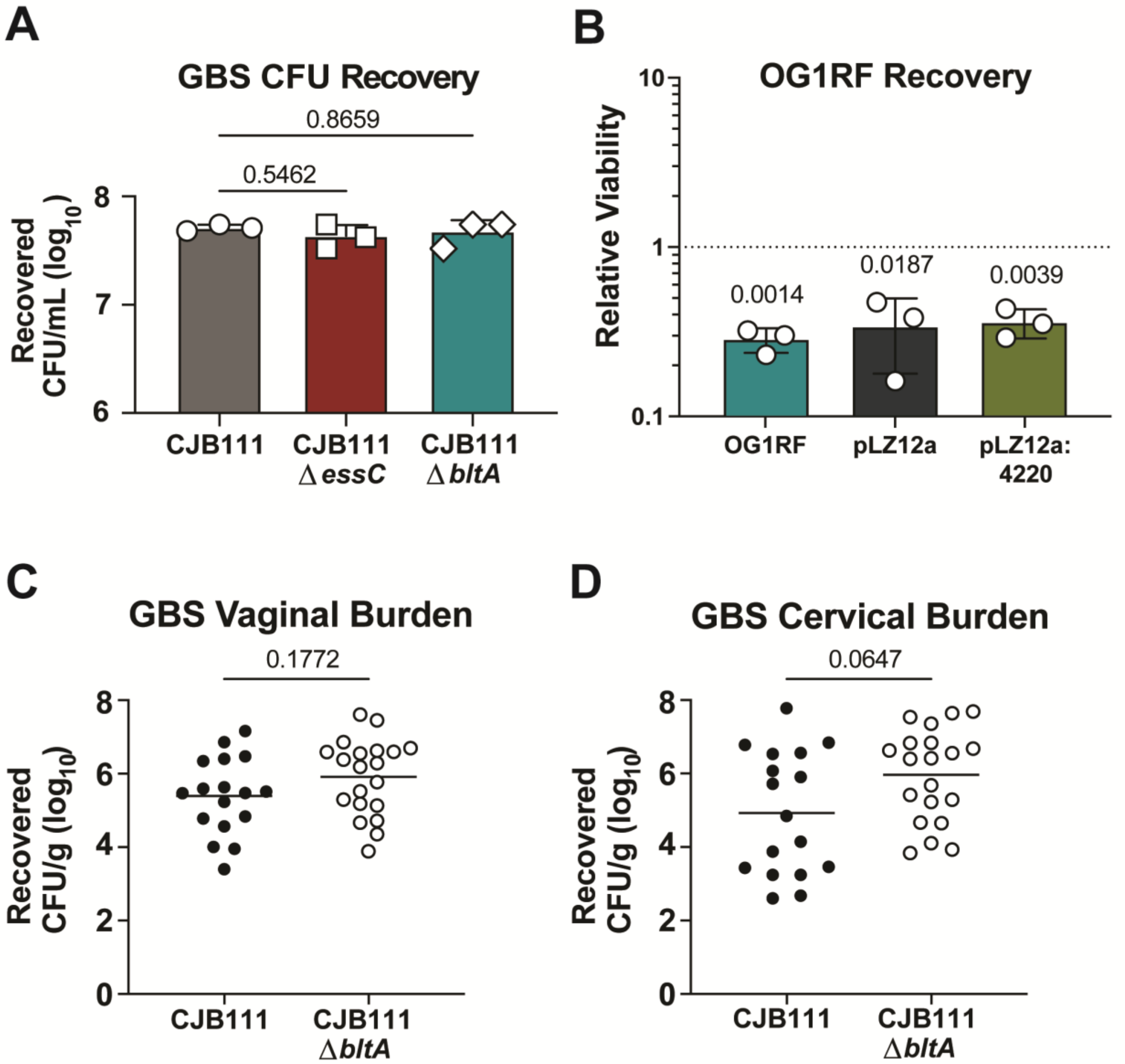

SFig. 3. GBS CFU recovery during *in vitro* and *in vivo* competition assays using the Δ*bltA* mutant. A) Parental CJB111, CJB111Δ*essC* mutant, and CJB111Δ*bltA* mutant CFU recovery following *in vitro* predator-prey inter-species competition experiments performed in **Fig. 2B**. Statistics reflect ordinary one-way ANOVA with Tukey's multiple comparison test. Data represent the mean of three independent experiments and error bars represent standard deviation. **B)** Predator-prey interbacterial competition assay performed between parental CJB111 or Δ*essC* mutants and *E. faecalis* OG1RF expressing no vector, empty vector pLZ12a, or pLZ12a:4220. Prey relative viability is calculated as in **Fig. 1A**. Statistics reflect one-sample t-tests against a hypothetical value of 1. Data represent the mean of at least three independent experiments and error bars represent standard deviation. GBS CFU burden recovered from the **C)** vagina and **D)** cervix during *in vivo* co-colonization competition experiments performed in **Fig. 2D-E**. Each dot represents one mouse and data from two independent experiments are combined in these figures (n = 17, 20 total in parental and Δ*bltA* mutant co-colonization groups, respectively). The bars in these plots show the median and statistics represent the Mann Whitney U test.

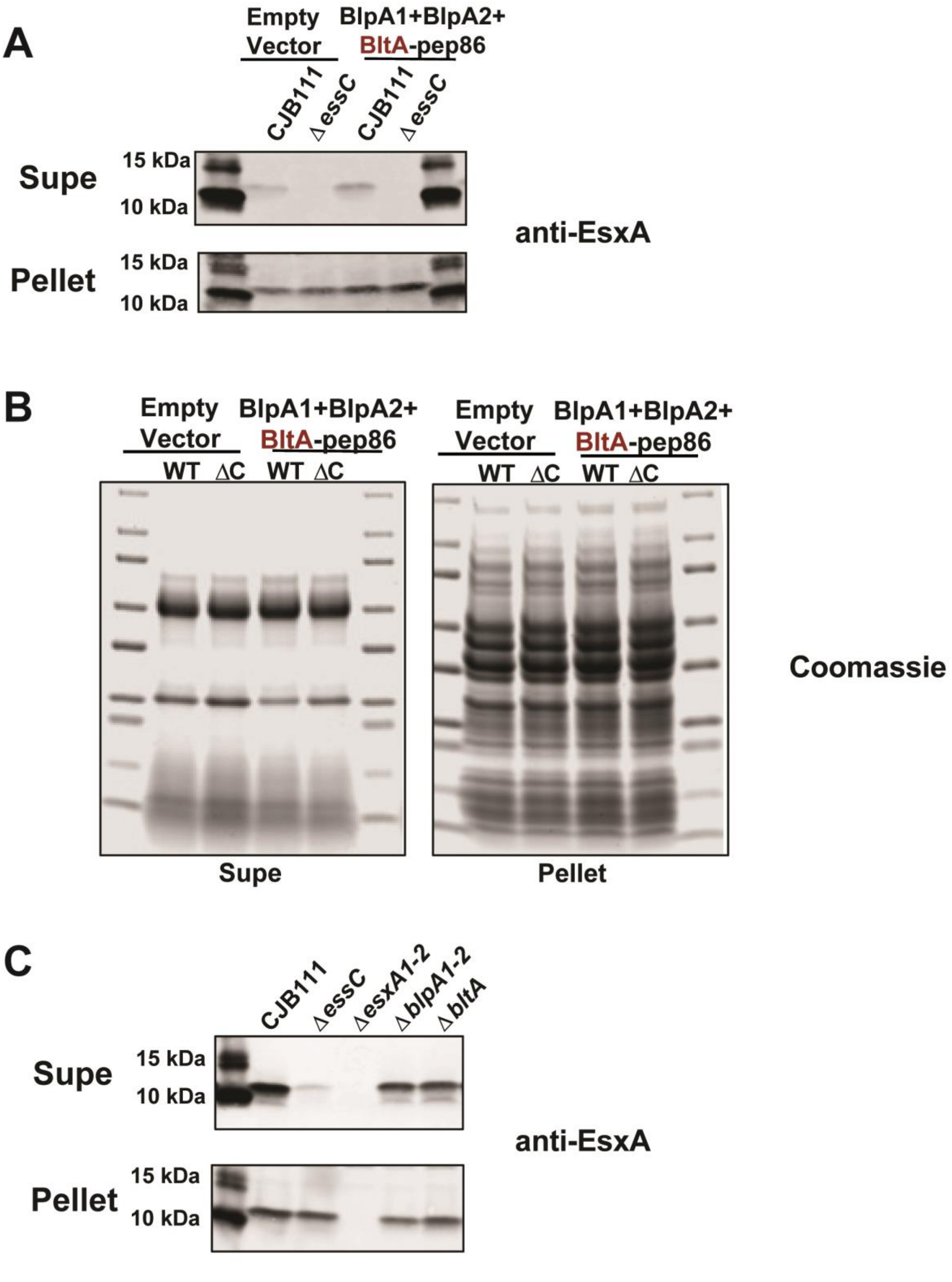

SFig. 4. Impact of EsxA secretion upon overexpression of BltA-pep86 or loss of BltA or BlpA1-2. A) Western blot showing EssC-dependent secretion of EsxA from subtype I CJB111 strains overexpressing BlpA1+BlpA2+BltA-pep86 compared to CJB111 empty vector controls. **B)** Coomassie-stained SDS PAGE gel indicating that wells were equally loaded for the western blots shown in **Fig. 3B** and **SFig. 4A. C)** Western blot showing equivalent EsxA secretion from CJB111, CJB111Δ*bltA*, and CJB111Δ*blpA1-2* strains. Blots pictured are representative of 3 independent experiments.

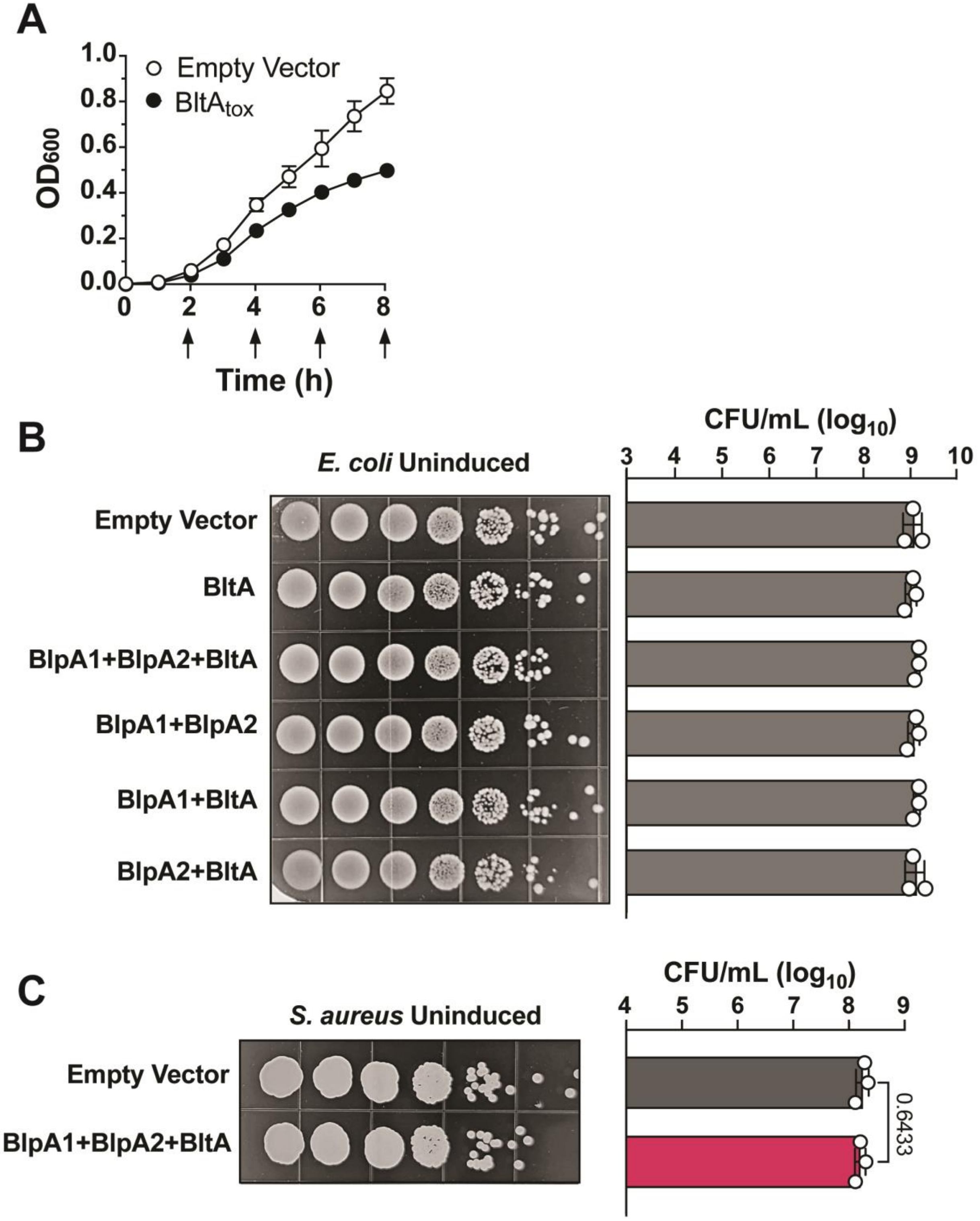

SFig. 5. Controls for BltA intoxication experiments in *E. coli* and *S. aureus*. A) Growth in liquid medium (as measured by OD_{600}) of *E. coli* expressing an empty vector control or an inducible plasmid containing the putative toxin domain of BltA. Arrows indicate addition of rhamnose inducer. Data represent the mean of three independent experiments and error bars represent standard error of the mean. Growth of **B)** *E. coli* and **C)** *S. aureus* intoxication strains from **Fig. 4C-D** on solid agar media not containing inducer. Serial dilutions are shown in the representative image on the left and results from three independent experiments are quantified in the panel on the right. Data represent the mean of three independent experiments and error bars represent standard deviation. Statistics in panel **C** reflect the student's t test.

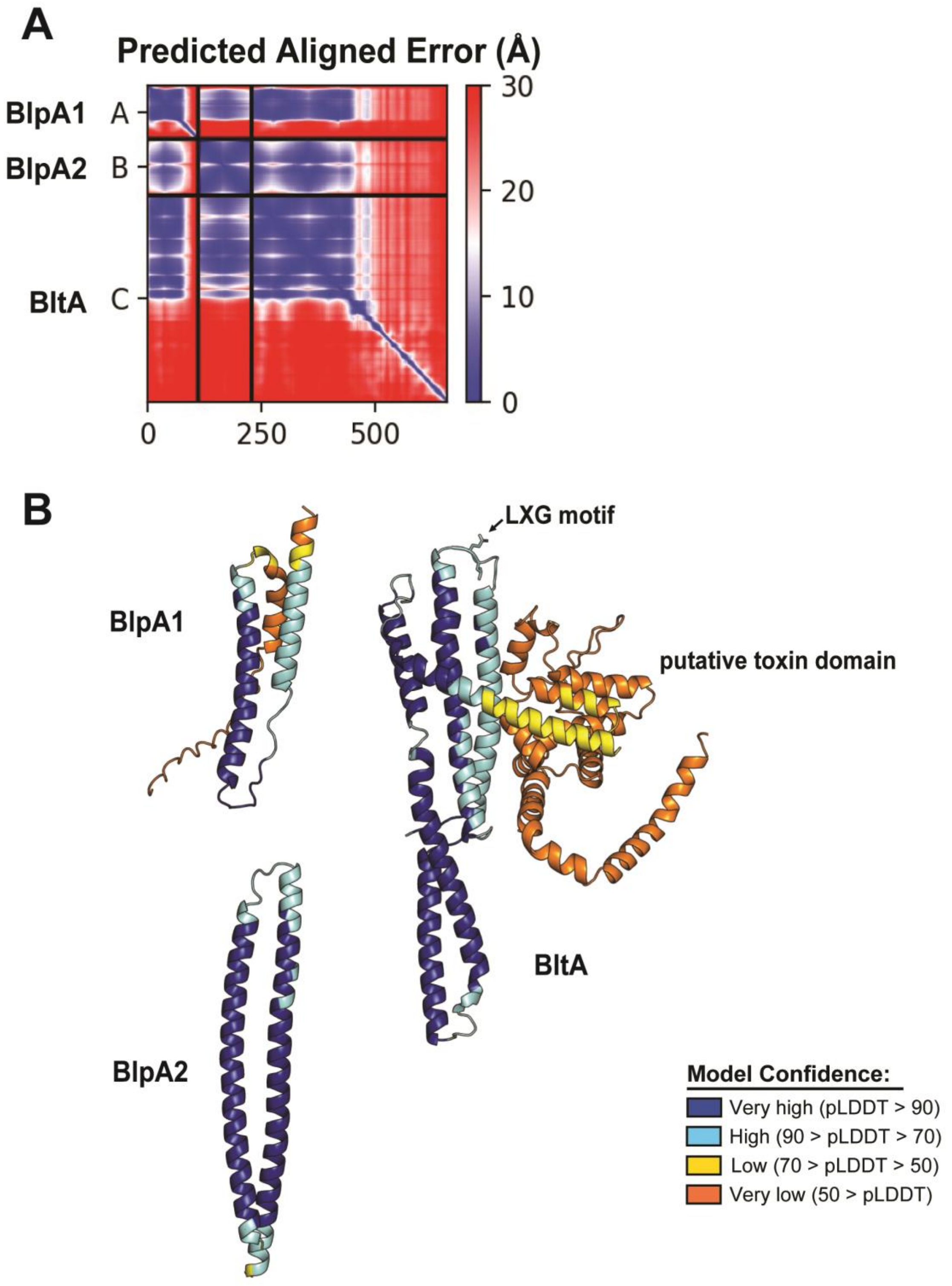

SFig. 6. Confidence scores and predicted aligned error for BlpA1-BlpA2-BltA AlphaFold predicted models in Fig. 5A. A) Predicted aligned error for the BlpA1 + BlpA2 + BltA complex. Colors indicate the confidence of domain positions (higher predicted error in red, lower predicted error in blue). Chain numbers A, B, and C correspond with BlpA1, BlpA2, and BltA, respectively. **B)** Predicted BlpA1 + BlpA2 + BltA complex model with colors corresponding to per-residue confidence level (predicted local distance difference test [pLDDT] score 1-100; pLDDT > 90 are expected to be modelled to very high accuracy; pLDDT < 50 may indicate unstructured/disordered regions but should not be interpreted with confidence).

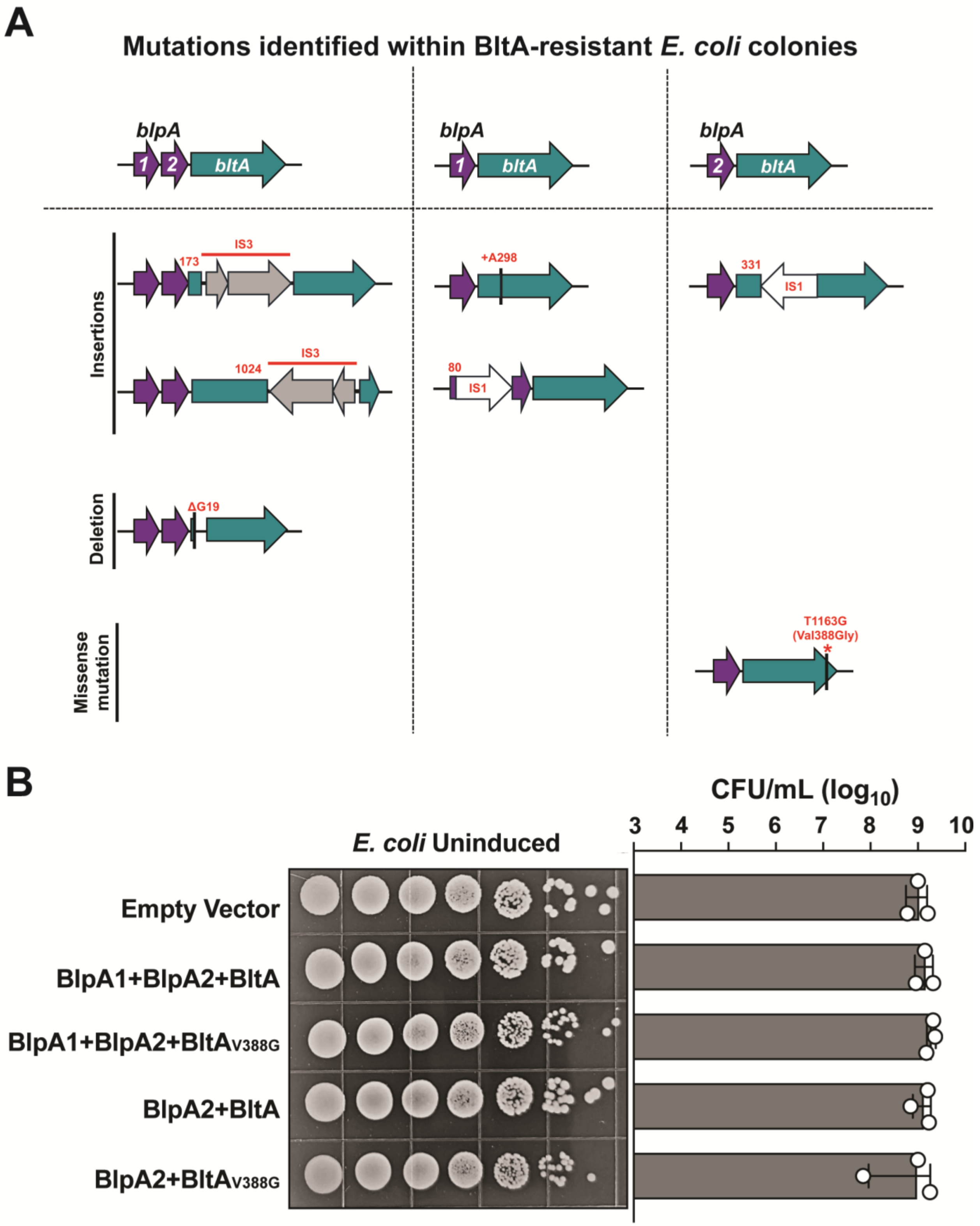

SFig. 7. Mutations identified in BltA-resistant *E. coli* colonies. A) Diagram of representative mutations from BltA-resistant *E. coli* colonies from strains carrying plasmids encoding BlpA1 + BlpA2 + BltA, BlpA1 + BltA, or BlpA2 + BltA. Mutations included insertion of IS1 and IS3 elements within all three genes on both the forward and reverse strands, a single nucleotide deletion in *bltA*, resulting in premature truncation of the BltA protein, a slip-strand insertion in *bltA* within a poly-A tract resulting in frameshift, and a BltA V388G missense mutation. Columns indicate the background in which resistant mutations were found. **B)** Growth of *E. coli* intoxication strains from **Fig. 5C** on solid agar media not containing inducer. Serial dilutions are shown in the representative image on the left and results from three independent experiments are quantified in the panel on the right. Data represent the mean of three independent experiments and error bars represent standard deviation.

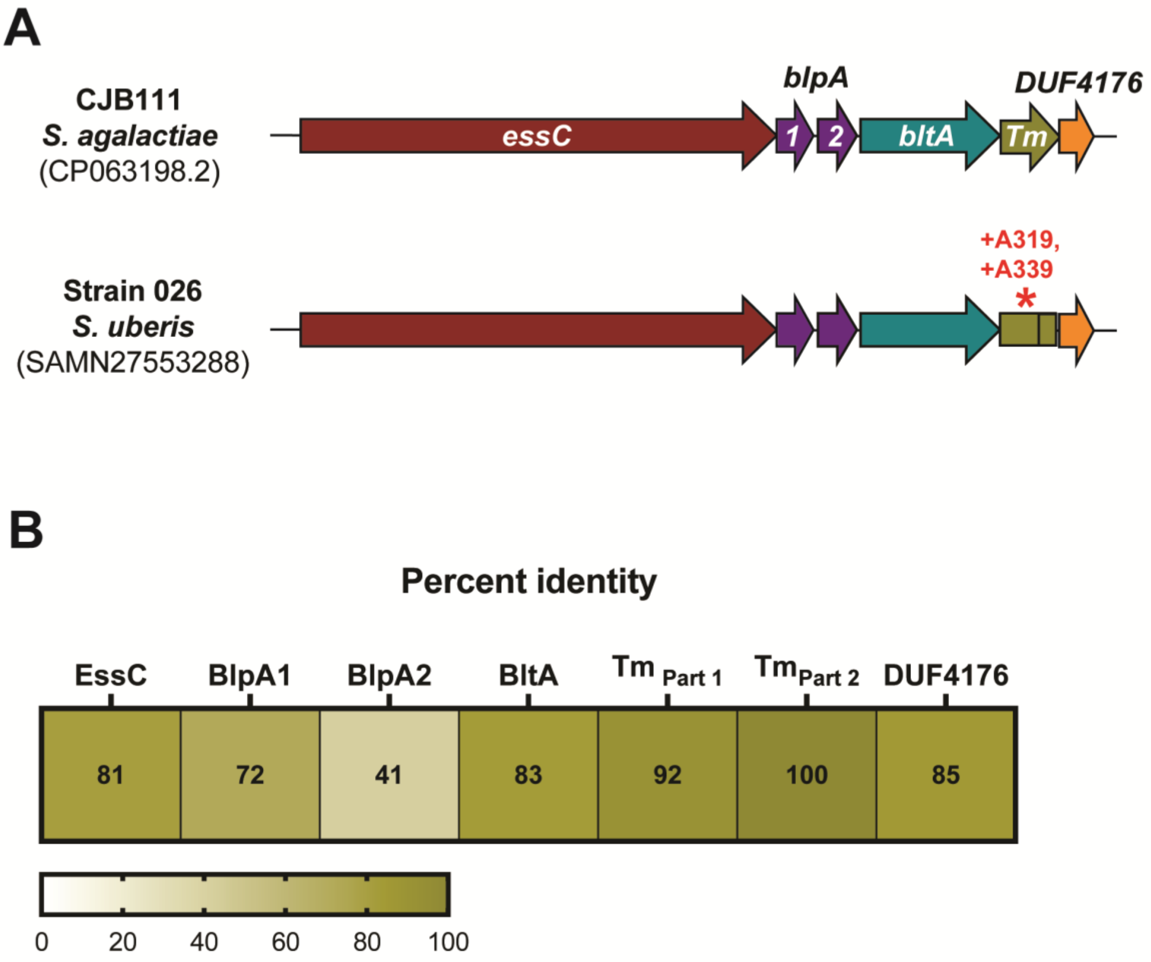

SFig. 8. GBS T7SS subtype I locus synteny with *S. uberis* T7SS. A) T7SS loci "LXG modules" representing GBS subtype I strain CJB111 (GenBank accession CP063198.2) and *S. uberis* Strain 026 (GenBank accession SAMN27553288). **B)** Heatmaps indicate homology of T7SS-associated proteins encoded for downstream of *essC* between GBS CJB111 (GBS T7SS subtype 1) and *S. uberis* Strain 026 via Clustal Omega analysis. Color intensity based on percent protein identity.
